# Identification and Validation of a m5C RNA Modification-Related Gene Signature for Predicting Prognosis and Immunotherapeutic Efficiency of Gastric Cancer

**DOI:** 10.1155/2023/9931419

**Published:** 2023-03-08

**Authors:** Li Song, Shouguo Wang, Qiankun Li, Yao Lu, Rungong Yang, Xianqi Feng

**Affiliations:** ^1^Academy of Advanced Interdisciplinary Studies, Qilu University of Technology, (Shandong Academy of Sciences), Jinan, Shandong 250353, China; ^2^Department of Tissue Repair and Regeneration, the First Medical Center of Chinese PLA General Hospital, Beijing 100853, China

## Abstract

**Background:**

5-methylcytosine (m5C) is a major site of RNA methylation modification, and its abnormal modification is associated with the development of gastric cancer (GC). This study aimed to explore the value of m5C-related genes on the prognosis of GC patients through bioinformatics.

**Methods:**

First, m5C-related genes were obtained by nonnegative matrix factorization (NMF) analysis and differentially expressed analysis. The m5C-related model was established and validated in distinct datasets. Moreover, a differential analysis of risk scores according to clinical characteristics was performed. The enrichment analysis was carried out to elucidate the underlying molecular mechanisms. Furthermore, we calculated the differences in immunotherapy and chemotherapy sensitivity between the high- and low-risk groups. Finally, we validated the expression levels of identified model genes by quantitative real-time polymerase chain reaction (qRT-PCR).

**Results:**

A total of five m5C-related subtypes of GC patients in the TCGA database were identified. The m5C-related model was constructed based on APOD, ASCL2, MFAP2, and CREB3L3. Functional enrichment revealed that the m5C-related model might involve in the cell cycle and cell adhesion. Moreover, the high-risk group had a higher abundance of stromal and immune cells in malignant tumor tissues and a lower tumor purity than the low-risk group. The patients in the high-risk group were more sensitive to chemotherapy and had better sensitivity to CTLA4 inhibitors. Furthermore, qRT-PCR results from our specimens verified an over-expression of ASCL2, CREB3L3, and MFAP2 in the cancer cells compared with the normal cells.

**Conclusion:**

A total of five GC subtypes were identified, and a risk model was constructed based on m5C modification.

## 1. Introduction

GC is the fifth most common malignancy worldwide and the third leading cause of global cancer-related mortality [1, 2]. Although clinical and surgical conditions improved significantly, the 5-year survival rate for GC patients remains very low, as more than 80% of patients are diagnosed at an advanced stage [3, 4]. Now, surgical resection is still the most effective treatment for early GC. Besides, chemotherapy, radiotherapy, immunotherapy, and molecular targeted therapy also play essential roles in the prognosis for GC [5, 6]. However, the mechanism of GC progression and metastasis is still unclear, and the prognosis leading to metastasis, recurrence, and advanced GC is not yet satisfactory. Therefore, it is urgent to study the mechanism of GC progression to develop new therapeutic strategies.

RNA modifications, such as N6 methyladenosine (m6A), play a visible role in epigenetic gene regulation and cell function and are closely related to many human diseases such as cancer, neurological diseases, and immune disorders [7–11]. As another important RNA modification, m5C has attracted more and more attention, and like m6A, m5C has its methyltransferase, demethylase, and binding proteins [12]. Members of the NOP2/Sun domain family 1-7 (NSUN1-7) and DNA methyltransferase (DNMT) homolog DNMT2 act as m5C writers in mammals and catalyze methylation at the C5 site of RNA [13, 14]. In contrast, TET2 oxidizes m5C to 5-hmC and then removes the methyl group [15, 16]. Subsequently, the Aly/REF output factor (ALYREF) and Y-box binding protein 1 (YBX1), which are characterized by readers, recognize and bind the m5C motif and then perform different biological functions [17, 18]. In addition, these regulatory factors are known to be synergistically involved in multiple tumor progressions with m5C modification. Chen et al. [19] found TRDMT1, an RNA methyltransferase known to methylate tRNA, is a writer of RNA m5C at sites of DNA damage and contributes to the resistance of cancer cells to radiotherapy and PARP inhibitors. Breast tumors expressing low levels of TRDMT1 are more responsive to radiotherapy. Du et al. [20] analyzed the clinical relevance of m5C regulators in pan-cancer. Liu et al. [21] wrote that the RNA m5C modification and its regulators have been shown to be involved in the progression of various cancers, including hepatocellular carcinoma, bladder cancer, glioblastoma multiforme, breast cancer, and head and neck carcinoma, indicating that RNA m5C might play an important role in tumorigenesis and progression.

In the present study, the effect of m5C on the prognosis of GC patients was explored by bioinformatics methods, which identified five m5C-related subtypes and mined four m5C-related genes as biomarkers, and based on the relationship of the prognosis model, patient survival, therapies, and the role of m5C in GC were demonstrated roundly.

## 2. Materials and Methods

### 2.1. Data Source

GC-related datasets were obtained from The Cancer Genome Atlas (TCGA) database (https://portal.gdc.cancer.gov/) and the Gene Expression Omnibus (GEO) database (https://www.ncbi.nlm.nih.gov/gds). The TCGA-GC dataset contains 32 normal cases and 373 cancer cases. The 345 cancer samples that have complete survival data were split into a training set (242 cases) and a testing set (103 cases) according to a ratio of 7 : 3. The *t*-test was used to compare the different characteristics between patients in training and testing sets. The results are shown in Table 1. Moreover, the GSE15459 dataset containing 192 cancer cases was obtained from the GEO database as a validation set. The 13 m5C RNA regulators (NOP2, NSUN2, NSUN3, NSUN4, NSUN5, NSUN6, NSUN7, DNMT1, TRDMT1, DNMT3A, DNMT3B, TET2, and ALYREF) were obtained from the previous literature [22].

### 2.2. Identification of m5C-Related Subtypes

373 GC samples from the TCGA database and the expression of 13 m5C RNA genes from the previous study were used for the nonnegative matrix factorization (NMF) analysis (R language, Version 0.23.0) [23] to identify m5C-related subtypes for GC patients. Then, overall survival (OS) and disease-specific survival (DSS) analyses of different subtypes were performed to screen the two subtypes with the most significant prognostic differences. These two subtypes were then used in subsequent analyses. Moreover, the clinical characteristics of the two subtypes were analyzed, and the results were visualized by ggplot2 (R package, Version 3.3.5) [24]. The immune cell infiltration of the two subtypes was calculated using the ssGSEA algorithm in the GSVA (R package) based on 24 immune cell types [25] and the MCPcounter algorithm by immunedeconv (R package, Version 2.0.4) based on 8 immune cell types and 2 stromal cell types.

### 2.3. Construction and Validation of an m5C-Related Model

The edgeR (R package) (Version 4.1) is used to perform differential expression analysis [26, 27]. *P* < 0.05 and |log2FC| > 1 were considered as a difference. The DEGs between the two subtypes with the most significant differences were detected, and the DEGs between the GC samples (*n* = 373) and para-cancerous samples (*n* = 32) in the TCGA dataset were also screened. By overlapping DEGs selected above, the DEm5CRGs were finally screened. Then, Cox regression analyses and the LASSO algorithm were adopted to construct the risk signature. The threshold was *P* < 0.05. The risk score of each sample was calculated by the following formula: risk score = *h*0(t) × exp (*β*_1_*X*_1_ + *β*_2_*X*_2_ + ... *β*_n_*X*_n_). The *h*0(*t*) was the baseline hazard function, and the *β* was the regression coefficient. GC patients in the training set were split into high- and low-risk groups based on the median risk score. At last, R package Survminer and survival ROC were used to plot the Kaplan–Meier (KM) and ROC curves to evaluate the risk model in the training set, and then the testing and validation sets were used to validate [28, 29].

### 2.4. Differential Analysis of Risk Values in Clinical Characteristics

The stratification survival analysis was performed to confirm whether the risk model could apply in different clinicopathological characteristics (including age, gender, radiotherapy, and chemotherapy). Meanwhile, the clinicopathological data were involved in variance analysis to investigate differences between clinicopathological features and risk values.

### 2.5. Construction of a Nomogram

The risk score and clinicopathological data were merged into Cox regression analyses to detect the independent prognosis factors. Then, the selected independent prognostic factors were integrated to establish a nomogram. Furthermore, the calibration curves and the decision curve analysis (DCA) were plotted to assess the nomogram.

### 2.6. Difference Analysis and GSEA

The DEGs between high- and low-risk groups were detected by the “limma” package. *P* < 0.05 and |log_2_^FC^| > 1 were considered as a difference. R package clusterProfiler (Version 4.0.2) was selected to perform GO enrichment and KEGG pathway analyses on these DEGs. Moreover, to further explore the related signaling pathways and potential biological mechanisms, R package clusterProfiler (Version 3.18.1) [30] was adopted to perform GSEA enrichment analysis. The significance thresholds for GSEA were |NES| > 1, *q* < 0.25, and NOM *P* < 0.05.

### 2.7. Analysis of Immunotherapy and Chemotherapy

The immune cell infiltration situations of the sample are inferred by the ESTIMATE and the CIBERSORT algorithms, and differences were analyzed between the high- and low-risk groups from the training set [31]. The tumor purity of the two groups was assessed using ABSOLUTE software. The expression of targeted immune checkpoints and the sensitivity to immunotherapy were analyzed in the two groups, and the prediction of susceptibility to PD-1 and CTLA4 inhibitors was analyzed in the two groups using the SubMap algorithm. We used oncoPredict (Version 0.2) in R language to analyze the sensitivity of commonly used chemotherapy drugs of GC samples [32].

### 2.8. Expression Validation of Prognostic lncRNAs

GC cell lines (MKN-27, MKN-45, and SMU-1) and human immortalized normal gastric cells CES-1 were obtained from CyberKang (Shanghai) Biotechnology Co., Ltd. and maintained in complete RPMI-1640 and DMEM medium (Welgene, Inc., Gyeongsan-si, Korea) at 37°C in a humidified 5% CO2 incubator. The prognostic gene expression levels were vilified by qRT-PCR. All cells were lysed with the TRIzol Reagent (cat.:356281), and total RNA was isolated. The RNA was reverse-transcribed to cDNA using the Script RT I First strand cDNA SynthesisAll-in-OneTMFirst-Strand cDNA Synthesis Kit (cat: G33330-50) before qRT-PCR. PCR was conducted in a BIO-RAD CFX96 Touch TM PCR detection system (Bio-Rad Laboratories, Inc., USA). The detailed forward and reverse primers are shown in supplementary table 1. All primers were synthesized by Servicebio (Servicebio, Wuhan, China). The experiment was repeated in triplicate on independent occasions.

### 2.9. Statistical analyses

The Wilcoxon test was used to perform a different comparison between the two groups. Associations between risk scores and gene function or related pathways were calculated by Pearson correlation.

## 3. Results

### 3.1. Identification of m5C-Related Subtypes

**Figure 1:**
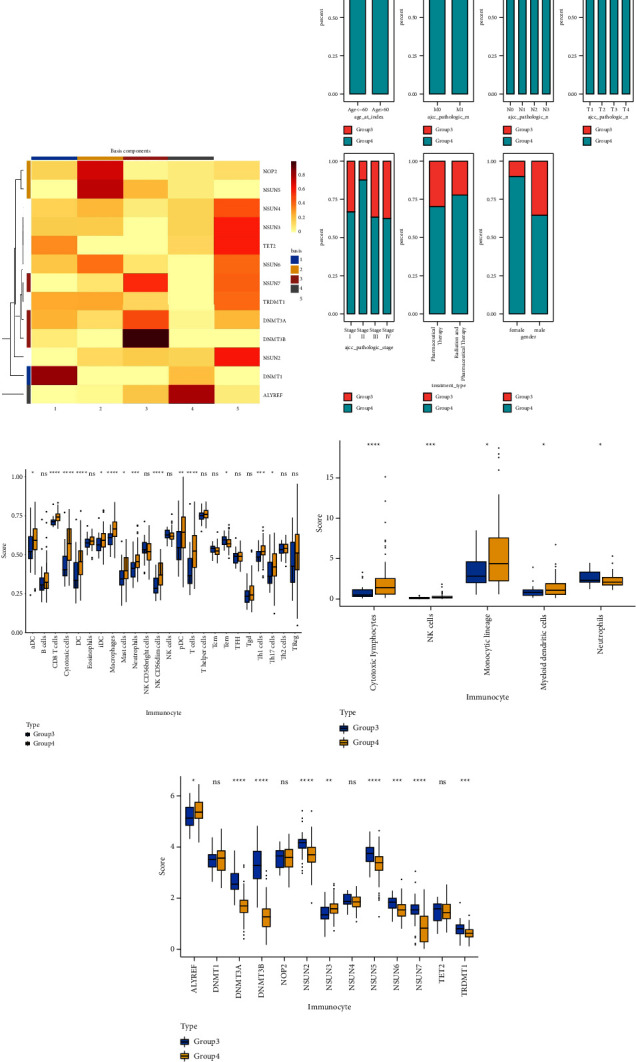
Identification of five m5C-related subtypes. (a) The heat map of m5C gene expression, the abscissa is the sample classification group, the ordinate is 13 m5C genes, and the lighter color indicated the smaller *P* value. (b) Differences in clinical features (age, AJCC pathologicM, AJCC _pathologic_N, AJCC _pathologic_T, AJCC _pathologic_stage, treatment_type, and gender) between subtypes. (c) Immune cell infiltration ssGSEA analysis of subtypes with significant survival differences. (d) Immune cell infiltration MCPcounter analysis of subtypes with significant survival differences. (e) the m5C gene expression pattern of significant survival differential subtypes. Group 1–5: 5 different subtypes by the rank value analyzed by NMF. ^*∗*^ represent *P* < 0.05, ^*∗∗*^ represent *P* < 0.01, ^*∗∗∗*^ represent *P* < 0.001, ^*∗∗∗∗*^ represent *P* < 0.0001.

NMF analysis finally identified five m5C-related subtypes (Figures S1 and 1(a)). OS and DSS analyses showed that survival differences between group3 and group4 were the most significant (*P* < 0.05; Figure S2). The distribution features of the clinical characteristics and the infiltration of immune cell types in group3 and group4 are shown (Figures 1(b) and 1(c)). The two groups were quite different in 5-cell concentrations (Figure 1(d)). Nine 5mC genes were significantly different between them (Figure 1(e)).

### 3.2. Construction and Validation of an m5C-Related Model

**Figure 2:**
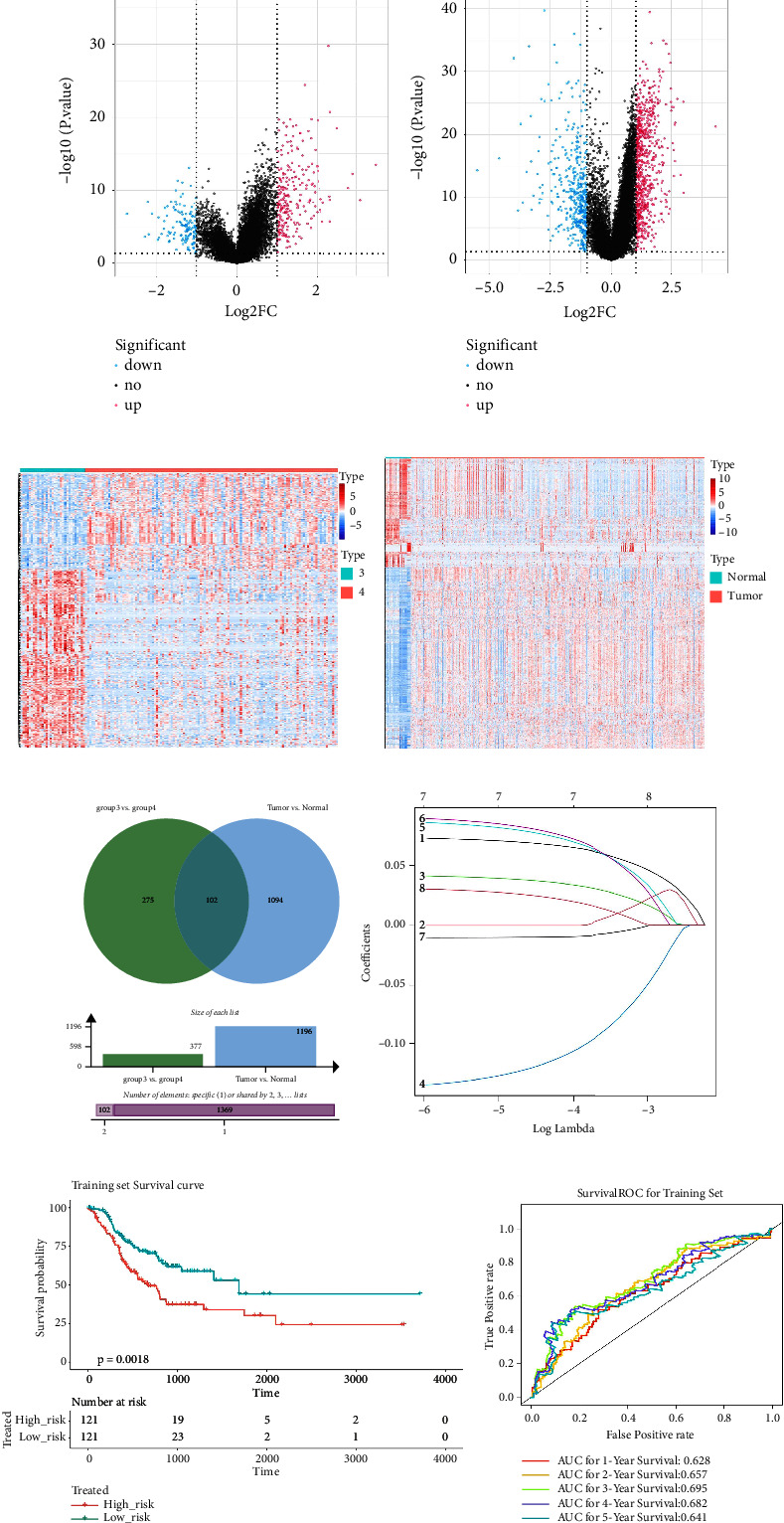
Construction of an m5C-related risk model. (a) The volcano map of DEGs of two subtypes of survival differences in GC. The abscissa represents log2^FC^, and the ordinate represents −log10 (adjust. *P* value). Red: upregulation; blue: downregulation. (a) The volcano map of DEGs between GC and normal samples from the TCGA database. Red: upregulation; blue: downregulation. (c) The heat map of DEGs of two subtypes. (d) The heat map of DEGs between normal and GC samples. (e) The venn diagram of DEGs in (a) and (b). (f) Screening characteristic genes by LASSO regression analysis. The abscissa is log (Lambda), and the ordinate is the coefficient of the gene. (g) The KM survival curve of high-andlow-risk groups in the training set. (h) The ROC curve and AUC for four DEm5CRGs.

In group3 and group4, 377 DEGs (245 up, 132 down) were identified (Figure 2(a)). In contrast, a total of 1196 DEGs (748 up and 448 down) were identified from normal and GC samples (in the TCGA dataset) (Figures 2(b)–2(d)). Finally, 102 DEm5CRGs were extracted from the intersection (Figure 2(e)). Cox regression (univariate) analysis showed that 8 DEm5CRGs were related to OS (*P* < 0.05; Table 2). Subsequently, a model involving 4 DEm5CRGs (APOD, ASCL2, MFAP2, CREB3L3) was constructed by LASSO and Cox regression (multivariable) analysis (Table 3 and Figure 2(f)). Then, the risk score of each sample was calculated with the following equation: risk score = 0.0807 × expAPOD + (0.1439) × expASCL2 + 0.1296 × expMFAP2 + 0.1091 × expCREB3L3, and the samples were grouped according to the median risk score. The high scores patients had a shorter OS (Figure 2(g)). The AUCs were 0.628, 0.695, and 0.641 (1, 3, and 5 years) (Figure 2(h)). The results showed that MFAP2, APOD, and CREB3L3 were highly expressed in the high score group, while ASCL2 was low. Similarly, the 103 GC (testing set) cases were split into high- (*n* = 52) and low-score (*n* = 51) groups, and the 192 GC cases (validation set) were split into high- (*n* = 96) and low-score (*n* = 96) groups. Results are consistent with the training set (Figures S(3a) and (3b)). The AUCs of the testing set were 0.670, 0.658, and 0.869 (1, 3, 5-year) (Figure S3(c)), and the AUCs of the validating set were 0.627, 0.671, and 0.700 (for 1, 3, 5-year OS) (Figure S3(d)).

The risk scores, patient survival status, survival time, and gene expression pattern are shown in Figures S4(a)–S4(c).

### 3.3. Differential Analysis of Risk Values

**Figure 3:**
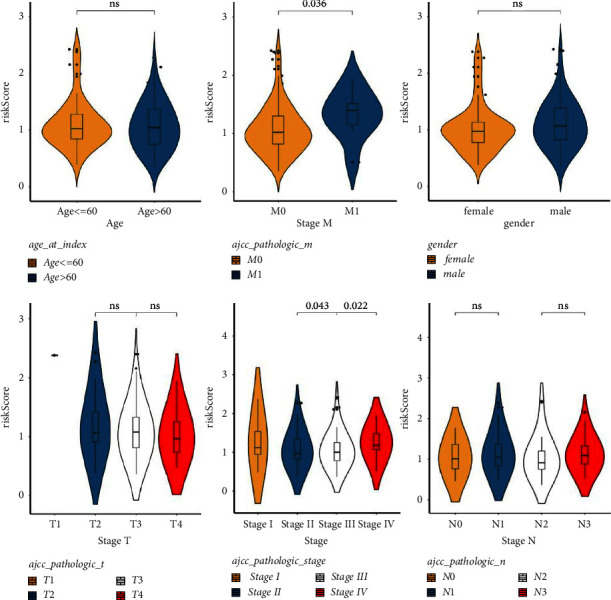
Analysis of differences in risk values in clinical features. The violin plot showed the analysis of differences in risk values under clinical characteristics, the abscissa represents different clinical traits, and the ordinate represents risk value scores.

To implore the clinicopathological characteristics and the survival of cases in the two groups, a hierarchical analysis of the km curve in the TCGA cases was performed. High score patients younger than 60 years old or whose pathological stage were T3 or T4 had a worse prognosis (Figure 3). Differences analysis between clinicopathological features and risk values showed that M0 and M1 and Stage II, Stage III, and Stage IV had significant differences (Figure S5).

### 3.4. Construction of a Nomogram

**Figure 4:**
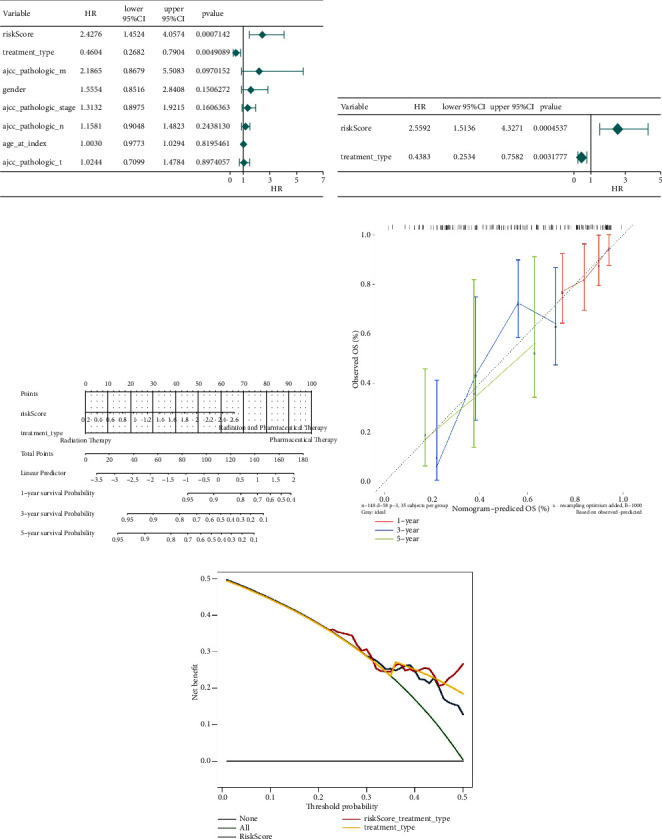
Establishment and validation of the prognosis model. (a) The forest map of univariate COX-independent prognosis. The hazard Ratio (HR) is the risk ratio. The lower 95% CI and the upper 95% CI are the 95% confidence intervals of risk values. (b) The forest map of multivariate COX-independent prognosis. (c) Nomograms for risk models and treatment types. (d) The correction curve of 1–5 years of clinical characteristics (*P* < 0.05). (e) The decision-making curve of 1–5 years of clinical characteristics (*P* < 0.05).

Score and treatment type were associated with GC cases prognosis and were the factor that were independent prognostic (Figures 4(a) and 4(b)). Then, the nomogram model was constructed to predict the survival of GC patients (Figure 4(c)). The calibration curves (C-index = 0.6547) and DCA curves of the nomogram were also plotted (Figures 4(d) and 4(e)).

### 3.5. Difference Analysis and GSEA

**Figure 5:**
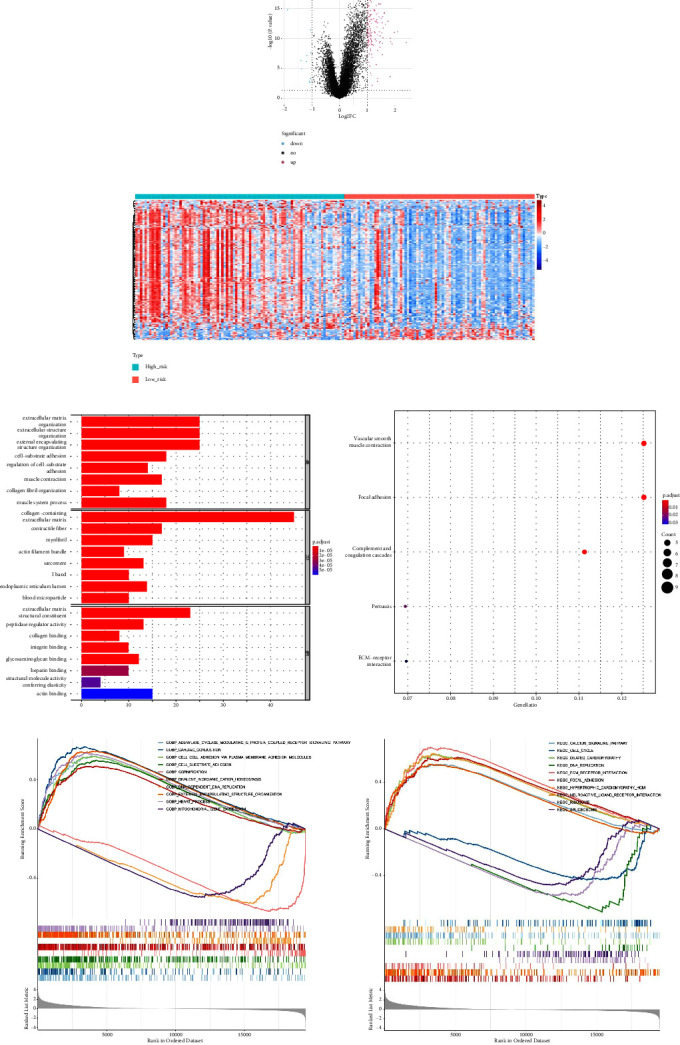
Differences analysis and the GO and KEGG pathway enrichment analysis of the 151 DEGs. (a) The volcano map of the DEGs, including 139 up-related genes (red) and 12 down-related genes (blue). (b) The heat map of the DEGs. (c) The top 8 terms are enriched in the GO systems, including extracellular matrix organization, extracellular structure organization, and external encapsulating structure organization. (d) KEGG enrichment analysis of DEGs, including 5 KEGG pathway, complement and coagulation cascades, vascular smooth muscle contraction, focal adhesion, pertussis, and ECM-receptor interaction. (e) GSEA enrichment analysis in high- and low-risk groups GO enrichment analysis. (f) GSEA enrichment analysis in high- and low-risk groups KEGG pathway analysis.

A total of 151 DEGs (139 up and 12 down) were identified (Figures 5(a) and 5(b)).

The main enriched cellular functions and KEGG pathway of DEGs between high- and low-risk groups are extracellular matrix organization, complement and coagulation cascades, ECM-receptor interaction, and so on (Figures 5(c) and 5(d)).

The results of GSEA analysis showed that the expression of focal adhesion, etc. were up-regulated (Figures 5(e) and 5(f)).

### 3.6. Analysis of Immunotherapy and Chemotherapy

**Figure 6:**
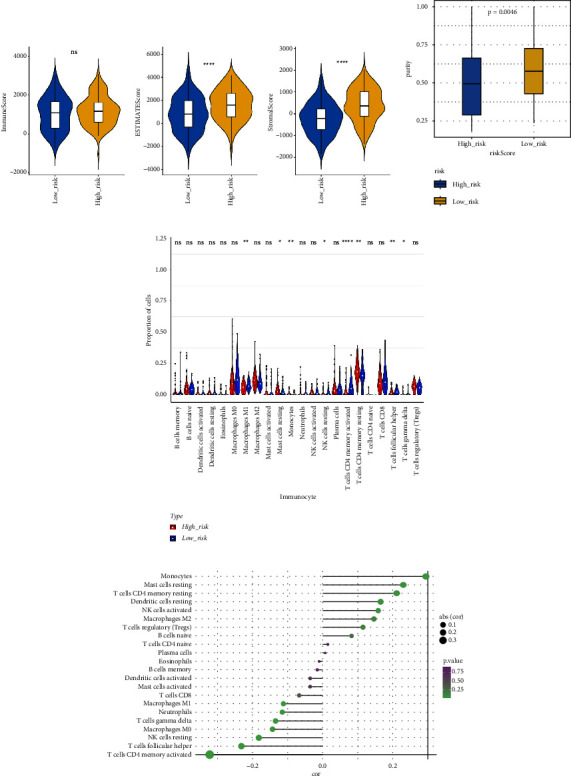
Treatment analysis of the high- and low-risk groups. (a) ESTIMATE difference scores for the high- and low-risk groups, based on the ESTIMATE comprehensive score and the matrix score. (b) Differences analysis in tumor purity between the high- and low-risk groups, having a significantly lower tumor purity of the high-risk group. (c) Immune cells proportion in the high- and low-risk groups, targeting tumor-infiltrating immune cells in each sample. (d) The lollipop chart depicting the correlation analysis of the risk value and immune cells.

The stromal score, the immune score, and the ESTIMATE composite score were obtained, and there were differences in the ESTIMATE composite score and the stromal score between high- and low-risk groups (Figure 6(a);  *P* < 0.0001). The high-risk group has lower tumor purity (Figure 6(b)). There are eight immune cell (macrophages M1, mast cells resting, etc.) abundances that differ between high- and low-risk groups (Figure 6(c)). The results of the correlation analysis between the risk score and immune cell abundance suggest that the abundance of monocytes, mast cells resting, and T cells CD4 memory resting was positively correlated with a risk score and the abundance of NK cells resting, T cells follicular helper, and T cells CD4 memory activated was negatively correlated with the risk score (Figure 6(d)).

**Figure 7:**
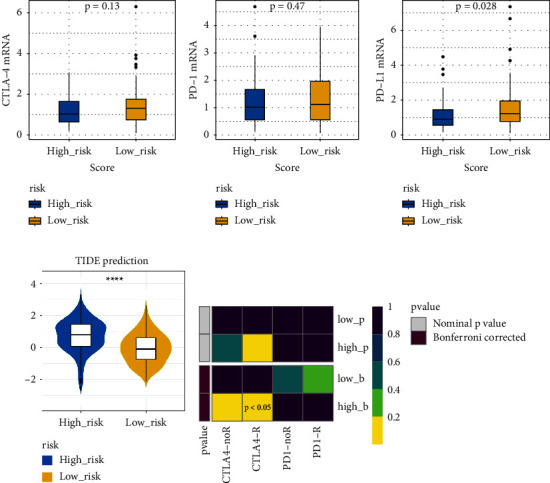
The TIED score and sensitivity scores of high- and low-risk groups. (a) Differences analysis of PD-1, PD-L1, and CTLA-4 in the high- and low-risk groups, basing on the TIED score of targeted immune checkpoints. (b) Sensitivity scores to whole immune checkpoints in high-low risk groups. (c) Sensitivity of the high- and low-risk groups predicted by the SubMap algorithm; R for immunotherapy responders and noR for immunotherapy nonresponders. *P* < 0.05, corrected by Bonferroni, was considered statistically significant.

The immune checkpoint PD-L1 expression levels differed significantly between high- and low-risk groups (Figure 7(a)). The expression of routine immune checkpoints in high- and low-risk groups is shown in supplementary table 2. The high-risk group was more sensitive to the overall immune checkpoint and had better sensitivity to CTLA4 inhibitors (Figure 7(b)).

Among 198 commonly used drugs for the treatment of GC, 182 species showed significant differences between high- and low-risk groups, and most high-risk groups were more sensitive to these drugs than low-risk groups (Figure 7(c)).

### 3.7. Expression Validation of Prognostic lncRNAs

**Figure 8:**
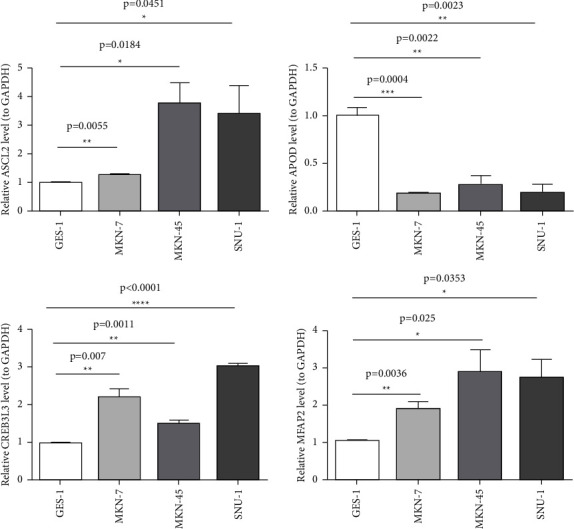
mRNA expression levels of the four prognostic genes in the GC cells and human immortalized normal gastric cells.

The qRT-PCR results from our specimens verified an over-expression of ASCL2, CREB3L3, and MFAP2 in GC cells compared with the human immortalized normal gastric cells (Figure 8).

## 4. Discussion

It is well known that GC is one of the leading causes of cancer-related deaths globally [33]. Although significant advancements in the treatments for GC have been acquired in recent years, the overall prognosis of GC patients is still poor [34]. m5C, in which the methyl group is attached to the fifth position of the cytosine ring, is catalyzed by RNA methyltransferase. m5C modification has also been closely related to cancer progression [35]. Meanwhile, bioinformatic studies have shown that m5C regulators could be used as a prognostic factor for lung adenocarcinoma (LUAD), head and neck squamous cell carcinoma (NHSCC), and hepatocellular carcinoma (HCC) [36–38].

With the development of molecular biology and clinical treatment with precision therapy, researchers have been exploring new prognostic markers of GC at the molecular level. Zhu et al. [3] revealed the expression, prognostic value, potential functional networks, protein interactions, and immune infiltration of MTFR2 (mitochondrial fission regulator 2) in GC, concluding that MTFR2 may be a potential prognostic marker and therapeutic target for GC patients. Zhu et al. [34] explored the association between VEGFR-2 and the prognosis of GC. They showed that the high expression of VEGFR-2 as well as the VEGFR-2 rs1870377 A > T genetic polymorphism may be prognostic factors for patients with resected GC. Zu et al. [39] considered that the preoperative prealbumin level was an independent prognostic factor for GC patients, and it is essential to predict the prognosis of patients with GC. Here, we established a prognosis model for GC based on five m5C-related subtypes and four DEm5CRGs (APOD, ASCL2, MFAP2, and CREB3L3) as biomarkers, employing 405 GC samples about second-generation sequencing data, clinical information, and copy gene variation information from the TCGA database, and at last, verifying the four biomarkers in GC cells compared with the human immortalized normal gastric cells by the RT-qPCR method, which is usually missing in bioinformatic analysis.

The four m5C-related genes based on 2 m5C-related subtypes affect the occurrence and development of cancer. Firstly, APOD (apolipoprotein D) is a lipocalin that participates in various cellular processes, including cytoprotection, and is a biomarker positively correlated with the prognosis of breast and prostate cancer [40]. APOD was also reported to be the prognostic factor of GC. Patients with high expression of APOD might have a shorter OS time, correlating with worse prognosis [41]. Second, ASCL2 (Achaete-scute homolog 2) is an essential helix-loop-helix transcription factor and a cancer stem cell marker, and specific reports have revealed that ASCL2 promotes cell proliferation and migration in colon cancer [42, 43]. In the meantime, ASCL2 also serves an essential role in the growth of GC. It was able to downregulate the expression level of miR223, contribute to EMT (the epithelial-mesenchymal transition), and promote gastric tumor metastasis, which indicated that ASCL2 might serve as a therapeutic target in the treatment of GC [44]. Third, MFAP2 (microfibril-associated protein 2) plays a vital role in the regulation of the integrin signal pathway in cancer cell-ECM (extracellular matrix) interaction. The intracellular form of MFAP2 can induce the transcription of integrin *α*4 in human osteosarcoma cell line SAOS-2 in vascular development [45]. Scholars also validated that MFAP2 was up-regulated in GC tissue, and it was implicated in the malignant behavior of GC cells, such as proliferation, migration, and invasion [46]. The fourth biomarker is CREB3L3, a member of the basic leucine zipper family and the AMP-dependent transcription factor family. It can link to acute inflammatory response and hepatocellular carcinoma [47]. Dewaele et al. illustrated that EWSR1-CREB3L3 gene fusion is associated with a mesenteric sclerosing epithelioid fibrosarcoma [48]. In GC, CREB3L3 is related to the OS derived from univariate and multivariate Cox regression analysis and is highly expressed in cancer tissues [49]. In a word, the four biomarkers can affect the occurrence and development of cancer in various degrees, including GC, and the guiding significance is great to analyze the relationship between the prognosis model and the survival of GC patients.

Moreover, GO and KEGG function analysis indicated that DEGs among the four gene biomarkers were closely correlated with biological processes and signaling pathways, such as ECM organization, extracellular structure organization, external encapsulating structure organization, complement and coagulation cascades, vascular smooth muscle contraction, and focal adhesion.

The m5C locus has been reported to be involved in a variety of biological processes, including structural stability and metabolism of RNA, tRNA recognition, and stress response [8]. A recent study has shown that in human urothelial cell carcinoma of the bladder, m5C regulators bound to the 3′UTR of oncogene mRNA, stabilizing its expression, thereby promoting cancer progression [50]. Yang et al. [17] found that NSUN2 (NOP2/Sun domain family, member 2; MYC-induced SUN domain-containing protein, Misu) was the main enzyme catalyzing m5C formation, while the Aly/REF export factor (ALYREF, an mRNA transport adaptor, also named THOC4) functioned as a specific mRNA m5C-binding protein regulating mRNA export. In addition, p57^Kip2^ was an important downstream gene regulated by NSUN2 in GC. p57^Kip2^ is the recently found CDK inhibitor of the Cip/Kip family and has been involved in many biological processes, including cell cycle control, differentiation, apoptosis, tumorigenesis, and development, which is in accordance with GO terms and KEGG pathways of 4 m5C-related genes [51, 52]. Previous studies found that the expression level of NSUN2 was negatively correlated with p57^Kip2^, and the ability of NSUN2 to knockdown cells proliferation was enhanced after p57^Kip2^ silencing in GC. It revealed another regulatory mechanism that NSUN2 plays an oncogenic role by repressing p57^Kip2^ expression in GC. The cause may be NSUN2 destabilizing the p57^Kip2^ mRNA relying on its methyltransferase activity and m5C modifications in the 3′-untranslated region (UTR) of p57^Kip2^ mRNA [53].

It has been reported that m5C modification is involved in immune microenvironment regulation, and the tumor immune microenvironment plays a role in the effect of m5C regulators on patient prognosis [54]. ALYREF, the Aly/REF nuclear export factor, functions as an m5C reader; its expression levels were significantly associated with immune infiltrating cells, such as B cells, macrophages, NK cells, and dendritic cells [55]. In an eight-lncRNAm5C-related prognostic signature, monocytes, memory B cells, activated mast cells, and naïve CD4 T cells presented a significant differences in high- and low-risk groups [56]. In the present study, significant differences existed in 5 types immune infiltrating cells obtained by the MCP counter algorithm, including NK cells, monocytic lineage, myeloid dendritic cells, cytotoxic lymphocytes, and neutrophils, which have similarities with previous studies.

## 5. Conclusion

Four DEm5CRGs were identified as biomarkers of the prognostic model in GC using three cohort profile datasets and integrated bioinformatics analysis. The expression pattern and prognostic value of m5C genes in GC were determined, and a novel m5C gene-based risk scoring system was established to predict the clinical outcomes of GC patients. It was found that m5C genes can reliably predict the OS of GC patients, providing a new target for the treatment of GC. However, to provide patients with a better prognosis and find the ideal individualized and targeted therapy, further prospective trials to test clinical efficacy are necessary.

## Figures and Tables

**Table 1:** Characteristics of patients in the training set and the testing set from the TCGA-GC cohort.

Characteristics	*n*	Training set	Testing set	*P* value
Total cases	148	102	46	
Age				
≤60	65	43	22	
>60	83	59	24	0.074
Metastasis				
M0	139	96	43	
M1	9	6	3	0.627
Node				
N0	30	20	10	
N1	46	31	15	
N2	32	23	9	
N3	40	28	12	**0.002**
Stage grouping				
Stage I	7	4	3	
Stage II	49	32	17	
Stage III	77	56	21	
Stage IV	15	10	5	0.310
Tumor				
T1	1	1	0	
T2	27	16	11	
T3	80	60	20	
T4	40	25	15	0.330
Treatment type				
Pharmaceutical therapy	86	58	28	
Radiation and pharmaceutical therapy	61	44	17	
Radiation therapy	1	0	1	0.385
Gender				
Female	51	35	16	
Male	97	67	30	0.250

Statistical significance is shown in bold.

**Table 2:** Cox regression (univariate) analysis 8 DEm5CRGs related to OS (*P* < 0.05).

Variable	HR	Lower 95% CI	Upper 95% CI	*P* value
APOD	1.1320	1.0342	1.2390	0.007148
GAMT	1.2210	1.0477	1.4229	0.010569
FKBP10	1.1510	1.0105	1.3111	0.034305
ASCL2	0.8947	0.8045	0.9949	0.039988
MFAP2	1.1789	1.0068	1.3804	0.040913
CREB3L3	1.1468	1.0051	1.3085	0.041760
PLEKHS1	0.8649	0.7516	0.9954	0.042918
AGT	1.1425	1.0013	1.3036	0.047765

CI: confidence interval.

**Table 3:** Cox regression (multivariable) analysis 4 DEm5CRGs as biomarkers.

Variable	coef	HR	Lower 95%CI	Upper 95%CI	*P* value
ASCL2	−0.1439	0.8659	0.7746	0.968	0.01136
APOD	0.0807	1.0840	0.9808	1.198	0.11401
CREB3L3	0.1091	1.1153	0.9691	1.284	0.12800
MFAP2	0.1296	1.1384	0.9601	1.350	0.13573

CI: confidence interval.

## Data Availability

The datasets generated and/or analyzed during the current study are available in the TCGA database (https://portal.gdc.cancer.gov/) and GEO database (https://www.ncbi.nlm.nih.gov/geo/).

## Supplementary Materials

Supplementary Materials Supplementary Figure 1: determination of the k value using the NMF rank survey with multiple parameters. Supplementary Figure 2: OS and DSS analyses of different subtypes. (a) Overall survival (OS) curves for 5 different subtypes. (b) Disease-specific survival (DSS) curves for 5 different subtypes. *P* < 0.05 showed statistically significant. Supplementary Figure 3: validation of the m5C-related risk model. (a) The KM survival curve of the high- and low-risk group in the validation set. (b) The KM survival curve of high- and low-risk group in the testing set. (c) The ROC curve and AUC for four signatures in the validation set. (d) The ROC curve and AUC of four signature in the test set. Supplementary Figure 4: the risk score, survival time, survival status, and expression of the four signatures in the training set (a), validation set (b), and testing set (c). Supplementary Figure 5: KM survival stratification analyses between high- and low-risk GC samples with clinicopathological data. Supplementary Table 1: the primer sequences for qRT-PCR. Supplementary Table 2: the detailed information for TIDE analysis.
