# Supplementary material for: Identification and Validation of a m5C RNA Modification-Related Gene Signature for Predicting Prognosis and Immunotherapeutic Efficiency of Gastric Cancer
Source: J Oncol. 2023 Mar 8;2023:9931419. doi: 10.1155/2023/9931419 (PMC10017215; doi:10.1155/2023/9931419)
Supplement: Supplementary Materials — Supplementary Figure 1: determination of the k value using the NMF rank survey with multiple parameters. Supplementary Figure 2: OS and DSS analyses of different subtypes. (a) Overall survival (OS) curves for 5 different subtypes. (b) Disease-specific survival (DSS) curves for 5 different subtypes. P < 0.05 showed statistically significant. Supplementary Figure 3: validation of the m5C-related risk model. (a) The KM survival curve of the high- and low-risk group in the validation set. (b) The KM survival curve of high- and low-risk group in the testing set. (c) The ROC curve and AUC for four signatures in the validation set. (d) The ROC curve and AUC of four signature in the test set. Supplementary Figure 4: the risk score, survival time, survival status, and expression of the four signatures in the training set (a), validation set (b), and testing set (c). Supplementary Figure 5: KM survival stratification analyses between high- and low-risk GC samples with clinicopathological data. Supplementary Table 1: the primer sequences for qRT-PCR. Supplementary Table 2: the detailed information for TIDE analysis. [file 9931419.f1.zip › Supplementary Figure 3.pdf]

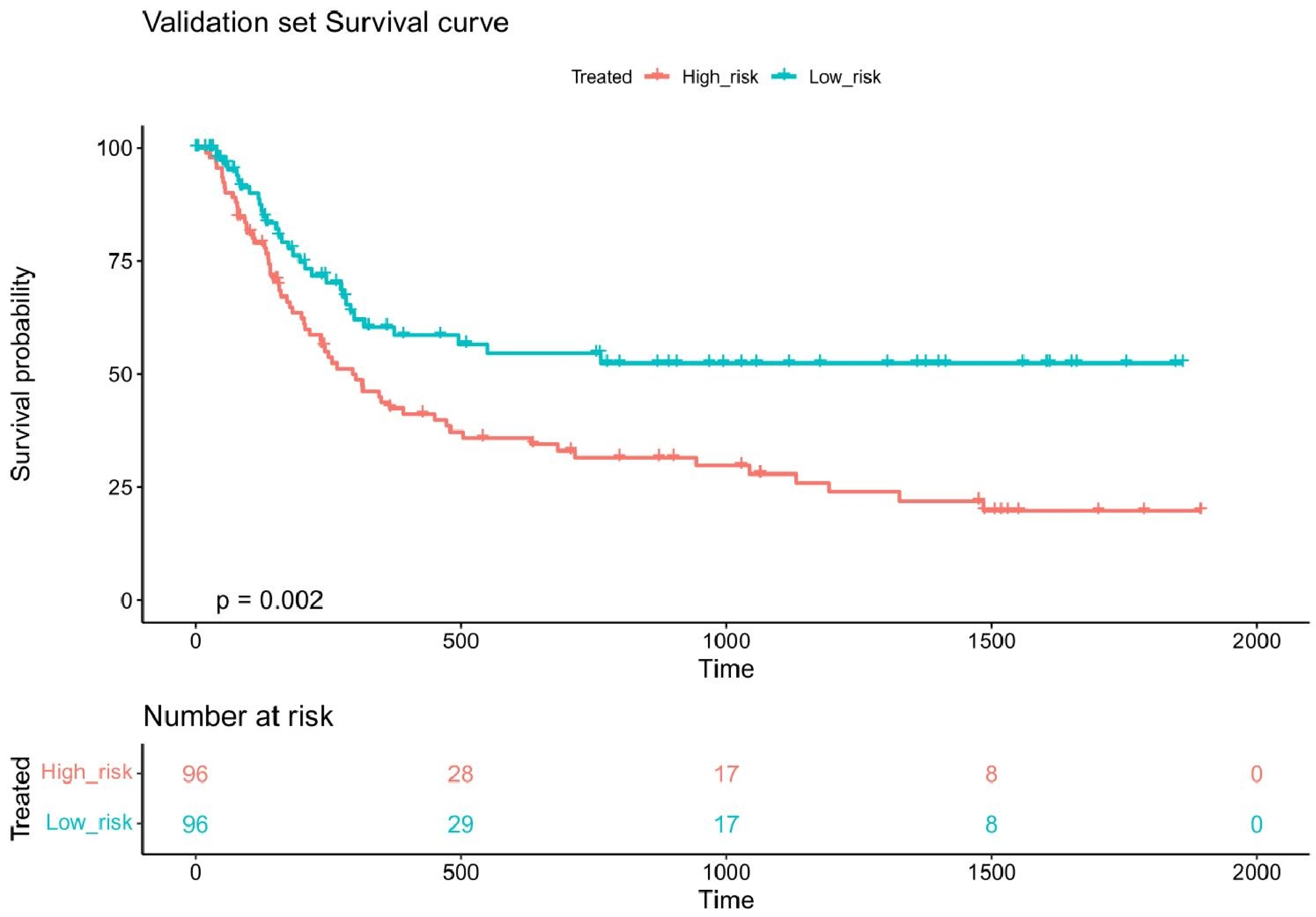
Validation set Survival curve
Treated High_risk Low_risk
Survival probability
100
75
50
25
0
p = 0.002
0 500 1000 1500 2000
Time
Number at risk
Treated
High_risk 96 28 17 8 0
Low_risk 96 29 17 8 0
0 500 1000 1500 2000
Time

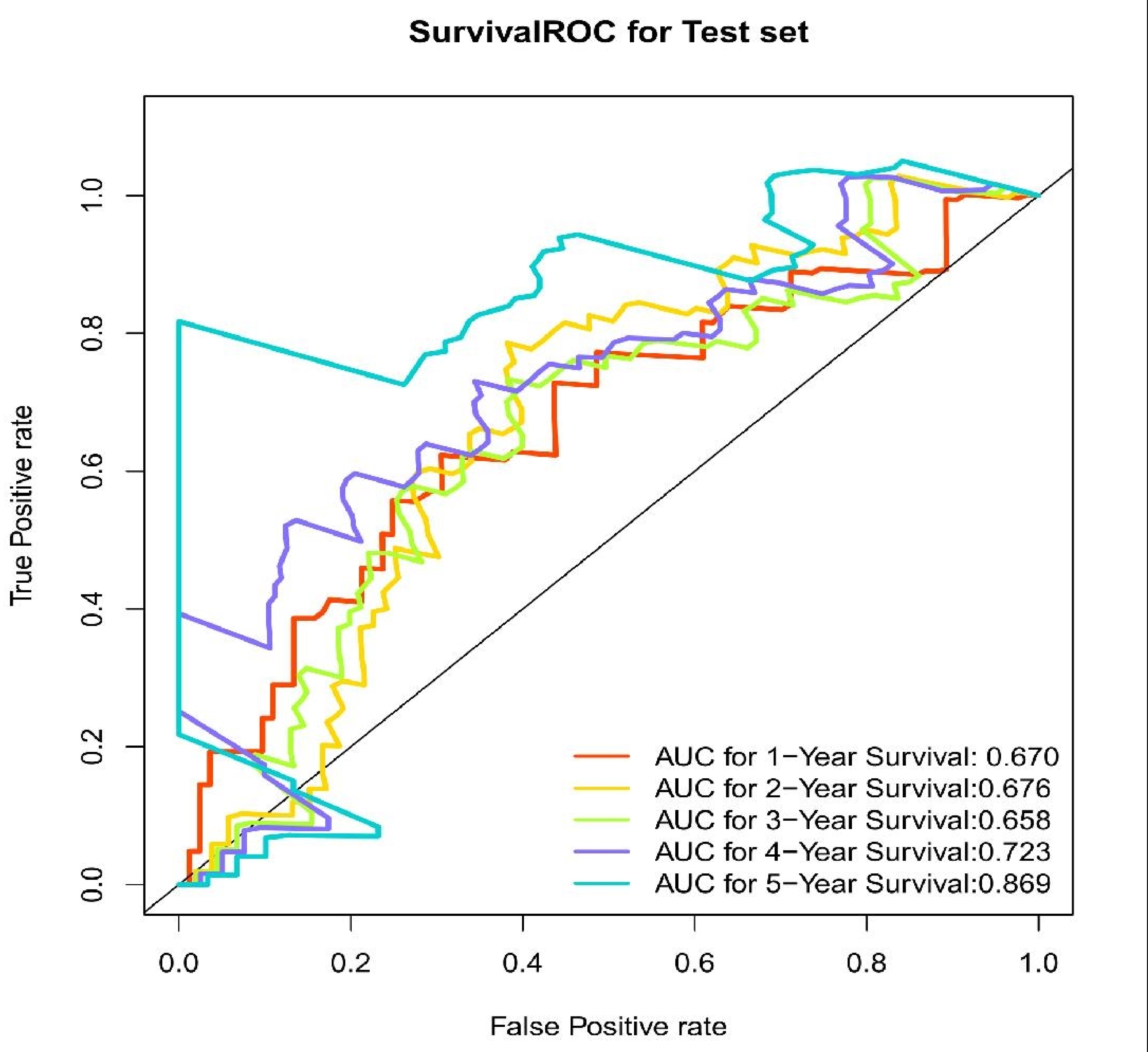
SurvivalROC for Test set
True Positive rate
False Positive rate
0.0 0.2 0.4 0.6 0.8 1.0
AUC for 1−Year Survival: 0.670
AUC for 2−Year Survival:0.676
AUC for 3−Year Survival:0.658
AUC for 4−Year Survival:0.723
AUC for 5−Year Survival:0.869

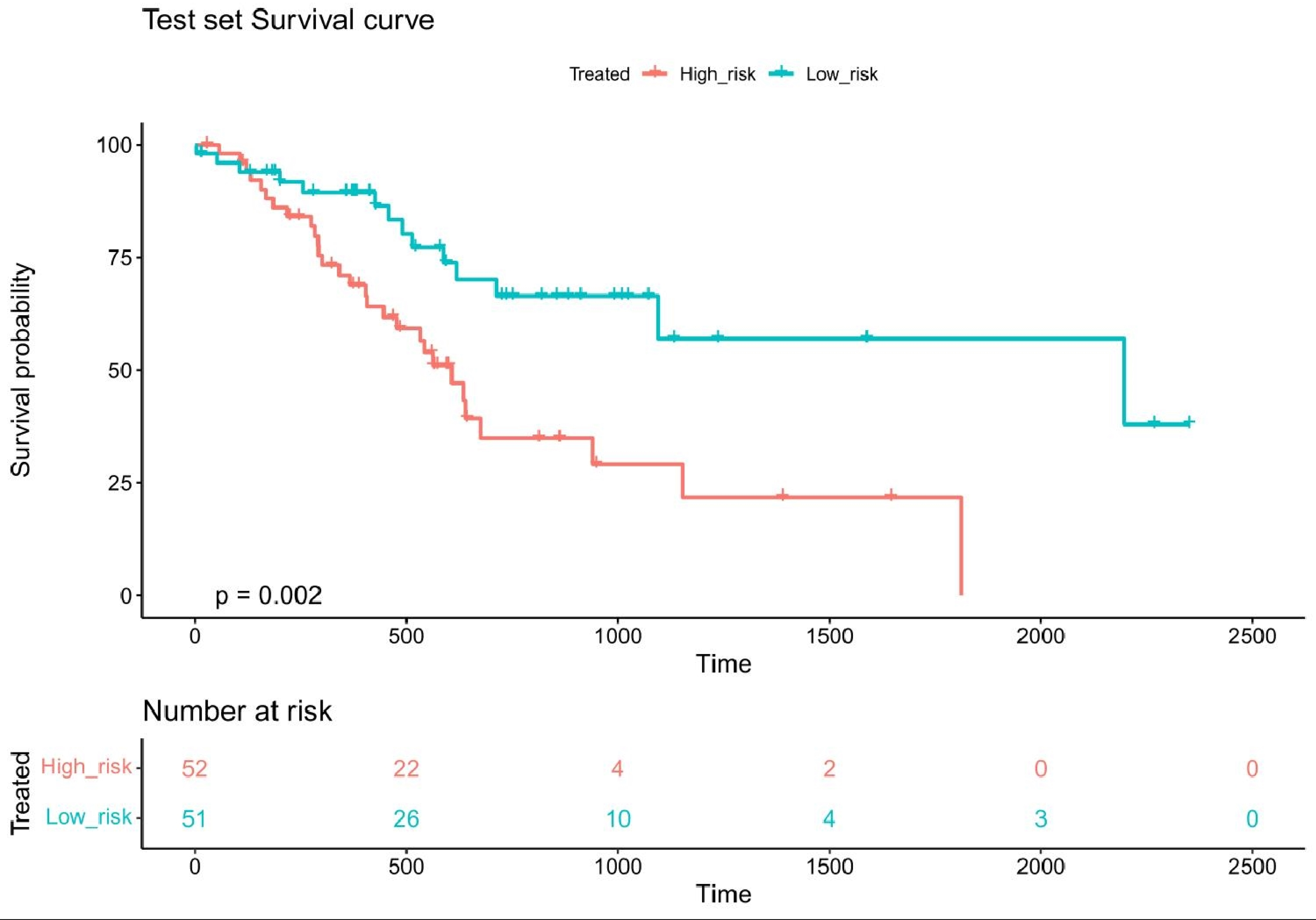
Test set Survival curve
Treated High_risk Low_risk
Survival probability
100
75
50
25
0
p = 0.002
0 500 1000 1500 2000 2500
Time
Number at risk
Treated
High_risk 52 22 4 2 0 0
Low_risk 51 26 10 4 3 0
0 500 1000 1500 2000 2500
Time

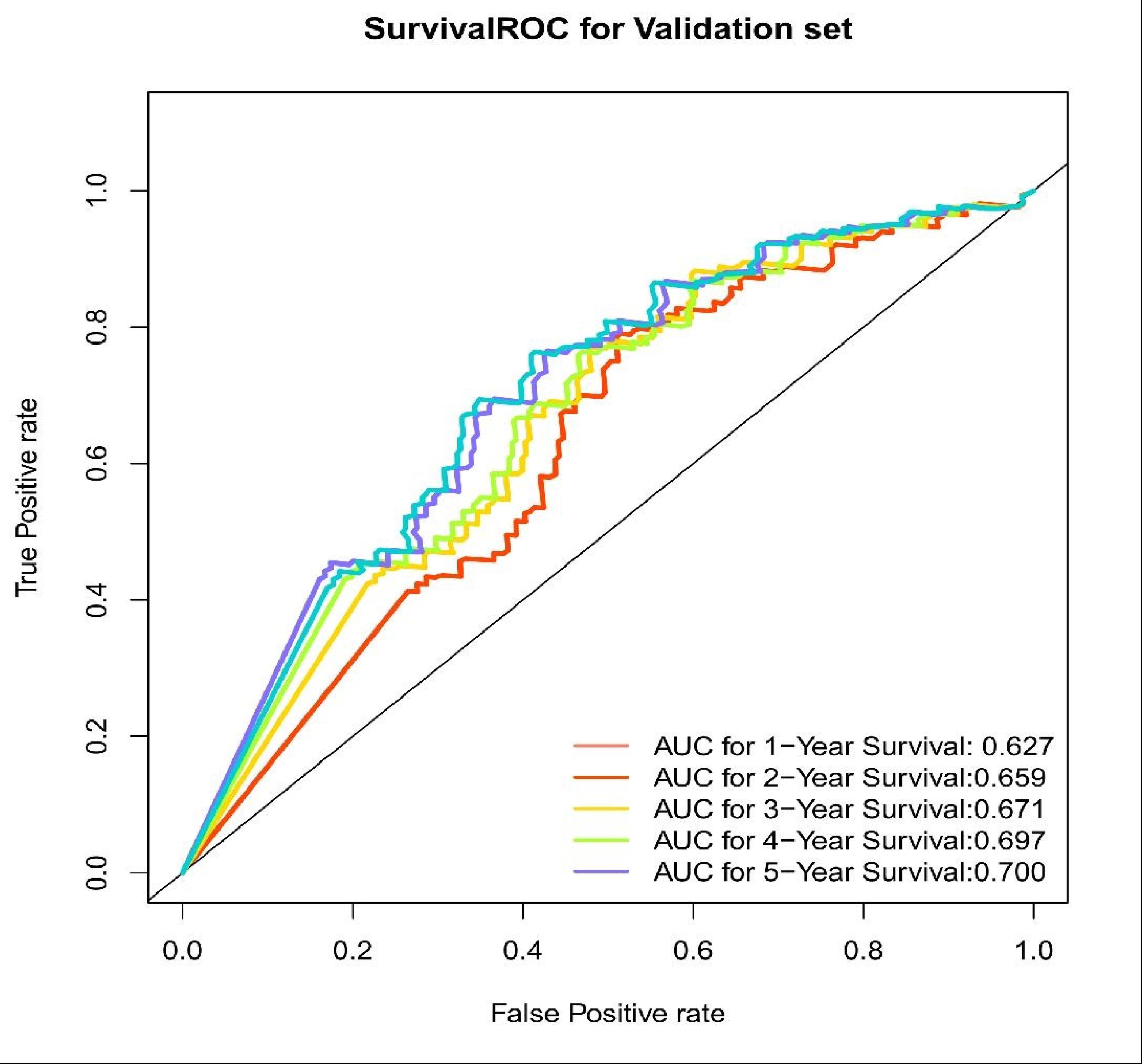
SurvivalROC for Validation set
True Positive rate
False Positive rate
0.0 0.2 0.4 0.6 0.8 1.0
AUC for 1−Year Survival: 0.627
AUC for 2−Year Survival:0.659
AUC for 3−Year Survival:0.671
AUC for 4−Year Survival:0.697
AUC for 5−Year Survival:0.700
