# Supplementary material for: Identification and Validation of a m5C RNA Modification-Related Gene Signature for Predicting Prognosis and Immunotherapeutic Efficiency of Gastric Cancer
Source: J Oncol. 2023 Mar 8;2023:9931419. doi: 10.1155/2023/9931419 (PMC10017215; doi:10.1155/2023/9931419)
Supplement: Supplementary Materials — Supplementary Figure 1: determination of the k value using the NMF rank survey with multiple parameters. Supplementary Figure 2: OS and DSS analyses of different subtypes. (a) Overall survival (OS) curves for 5 different subtypes. (b) Disease-specific survival (DSS) curves for 5 different subtypes. P < 0.05 showed statistically significant. Supplementary Figure 3: validation of the m5C-related risk model. (a) The KM survival curve of the high- and low-risk group in the validation set. (b) The KM survival curve of high- and low-risk group in the testing set. (c) The ROC curve and AUC for four signatures in the validation set. (d) The ROC curve and AUC of four signature in the test set. Supplementary Figure 4: the risk score, survival time, survival status, and expression of the four signatures in the training set (a), validation set (b), and testing set (c). Supplementary Figure 5: KM survival stratification analyses between high- and low-risk GC samples with clinicopathological data. Supplementary Table 1: the primer sequences for qRT-PCR. Supplementary Table 2: the detailed information for TIDE analysis. [file 9931419.f1.zip › Supplementary table 2 .docx]

Patient

TCGA -BR- 8059-01A- 11R-2343-13 TCGA -BR- 7957-01A- 11R-2203-13 TCGA -D7-6522-01A-11R -1802- 13 TCGA -BR- 6705-01A- 12R-1884-13 TCGA -CD-A487-01A-21R-A24K- 31 TCGA -BR- 7715-01A- 11R-2055-13 TCGA -BR-A4IV-01A- 31R-A251-31 TCGA -BR-A4J5-01A-21R-A251-31 TCGA -VQ-AA64-01A-11R -A414- 31 TCGA -CD- 8530-01A- 11R-2343-13 TCGA -MX -A663-01A-11R-A31P-31 TCGA -VQ-A8E2-01A- 11R-A36D- 31 TCGA -CD- 5798-01A- 11R- 1602-13 TCGA -B7-A5TN-01A-21R-A31P -31 TCGA -CD- 5803-01A- 11R- 1602-13 TCGA -BR- 6453-01A- 11R-1802-13 TCGA -BR- 7959-01A- 11R-2343-13 TCGA -EQ- 8122-01A- 11R-2343-13 TCGA -BR- 8297-01A- 12R-2343-13 TCGA -BR- 8289-01A- 11R-2343-13 TCGA -BR- 6454-11A-01R-1802-13 TCGA -BR- 8380-01A- 11R-2343-13 TCGA -FP-7998-01A-11R -2203-13 TCGA -VQ-A922-01A-11R -A414- 31 TCGA -CD-A489-01A- 11R-A24K- 31 TCGA -CG- 5734-01A- 11R- 1602-13 TCGA -FP-7735-11A-01R -2055-13 TCGA -VQ-A91A -01A-11R -A414- 31 TCGA -D7-8578-01A-21R -2343- 13 TCGA -BR- 7717-01A- 11R-2055-13 TCGA -FP-7829-11A-01R -2055-13 TCGA -CD- 5813-01A- 11R- 1602-13 TCGA -BR- 8364-01A- 11R-2343-13 TCGA -CG- 5719-01A- 11R- 1602-13 TCGA -BR- 6563-01A- 13R-2055-13

TCGA -BR- 6457-01A-21R-1802-13

No benefits Responder

| FALSE  TRUE  FALSE  FALSE  TRUE  TRUE  TRUE  FALSE  FALSE  TRUE  FALSE  FALSE  FALSE  FALSE  FALSE  FALSE  FALSE  TRUE  FALSE  TRUE  FALSE  TRUE  FALSE  FALSE  FALSE  FALSE  TRUE  FALSE  TRUE  TRUE  TRUE  FALSE  FALSE  FALSE  FALSE  FALSE | FALSE FALSE FALSE FALSE FALSE FALSE FALSE FALSE FALSE FALSE FALSE FALSE FALSE FALSE FALSE FALSE FALSE FALSE FALSE FALSE FALSE FALSE FALSE FALSE FALSE FALSE FALSE FALSE FALSE FALSE FALSE FALSE FALSE FALSE FALSE  FALSE |
| --- | --- |

TIDE IFNG

3.35

2.63

2.63

2.6

2.6

2.46

2.41

2.39

2.34

2.33

2.3

2.25

2.17

2.12

2.09

2.05

2.05

2

1.98

1.98

1.98

1.92

1.88

1.88

1.84

1.83

1.82

1.8

1.8

1.8

1.79

1.79

1.78

1.78

1.75

1.75

MSI Expr SigMerck18 CD274 CD8

-0.37 -0.47 0.22 -0.38 -0.35 -0.36 -0.29 -0.51 -0.82 -0.22 -0.61 -0.51 -0.15 -0.12 0.51 -0.27 -0.22 -0.49 -0.56 -0.55 0.05 -0.68 3.62 -0.65 -0.13 0.83 -0.12 0.4 -0.12 -0.35 -0.24 -0.05 -0.6 -0.39 -0.13

-0.32 -1.13 0.98 -0.61 -1.07 -1.12 -1.2 -0.98 -0.71 -1 -0.99 -0.06 -0.79 -0.1 1.64 0.07 -0.22 -1.41 -0.41 -1.06 -0.12 -1.07 3.57 -0.53 -0.43 1.15 -1.4 -0.96 -1.11 -1.09 -1.82 0.49 -0.64 -0.86 0.42

-0.01

0.72 0.27 0.43 0.08 0.36 0.06 0.32 0.27 0.04 0.05 0.06 0.01 0.72 0.16 0.21 0.61 0.04 0.03 0.44 0.11 0.92 0.69 0.14 0.01 0.15 0.8 0.96 0.05 0.04 0.47 0.93 0.04 0.15 0.2 0.33

0.14

-0.48 -0.56 1.59 -0.08 -0.68 -0.86 -0.59 -0.35 -0.83 -0.37 -0.71 -0.36 -0.06 -0.22 1.6 0.84 -0.4 -1.06 -0.5 -0.71 0.69 -0.61 2.47 -0.95 -0.27 1.29 -0.66 -0.68 -0.43 -0.57 -1.31 0.96 -0.12 -0.04 1

0.4

-0.46

CTL.flag

| -0.83  -0.28  1.7  -0.23  -0.71  -0.88  -0.73  -0.42  -0.68  -0.22  -0.55  0.3  0.04  -0.77  2.17  1.03  -0.23  -1.03  -0.3  -0.51  0.59  -0.52  3.03  -1.12  0.21  0.51  -0.41  -1.28  0.21  -0.05  -1.29  1.35  0.42  -0.75  1.87  0.48 | FALSE FALSE TRUE FALSE FALSE FALSE FALSE FALSE FALSE FALSE FALSE FALSE FALSE FALSE TRUE TRUE FALSE FALSE FALSE FALSE TRUE FALSE TRUE FALSE FALSE TRUE FALSE FALSE FALSE FALSE FALSE TRUE FALSE FALSE TRUE  TRUE |
| --- | --- |

Dysfunction Exclusion MDSC CAF

-0.26 3.35 0.13

0.26 2.63 0.02

-0.19 0.02 0.19 0.15 -0.02 -0.02 0.11 0.04 0.05 0.16 0 0.05 -0.08 -0.16 0.04 0.16 0.04 0.09 -0.15 -0.02 -0.2 0.14 0.01 -0.15 -0.02 0.04 -0.02 0.11 -0.03 -0.08 -0.1 -0.01 -0.16

2.63 1.35 -0.88 -0.47 0.21 0.5 -0.74 0.25 0.44 0.19 1.01 0.07 2.09 2.05 0.51 -0.38 0.08 -0.66 1.98 0.61 1.88 0.23 0.28 1.83 0.27 0.02 1.01 -0.03 0.18 1.79 1.03 1.46 1.75

1.75

-0.22 2.6 2.6 2.46 2.41 2.39 2.34 2.33 2.3 2.25 2.17 2.12 0.56 -1.13 2.05 2 1.98 1.98 -1.29 1.92 -2.03 1.88 1.84 -2.31 1.82 1.8 1.8 1.8 1.79 1 1.78 1.78 0.33

2.06

-0.04

TAM M2

0.04 0.02 -0.01 0.02 0.02 0.06 0.02 0.02 0.02 0.03 0.05 0.04 0.02 -0.01 -0.05 0.03 -0.03 0.02 0.03 0.08 -0.04 0.04 -0.12 0.01 0.02 -0.08 0.05 -0.04 -0.03 0.06 0.05 0 0.03 0.02 0.02

0.34 0.37 0.18 0.36 0.15 0.15 0.38 0.37 0.23 0.29 0.26 0.13 0.32 0.28 0.23 -0.04 0.31 0.12 0.23 0.12 0 0.28 0.03 0.13 0.24 -0.11 0.25 0.28 0.33 0.09 0.26 0.23 0.35 0.27 0.21

0.34

0.02

| TCGA -BR- 6852-01A- 11R-1884-13 | FALSE | FALSE | 1.7 | 3.08 | 0.99 | 2.25 | 2.09 | 2.66 | TRUE | 1.7 | -1.72 | -0.08 | -0.04 | -0.13 |
| --- | --- | --- | --- | --- | --- | --- | --- | --- | --- | --- | --- | --- | --- | --- |
| TCGA -CD-A48C-01A-11R -A24K-31 | FALSE | FALSE | 1.69 | 0.23 | 0.19 | -0.29 | -0.12 | 0.26 | FALSE | -0.77 | 1.69 | 0.13 | 0.12 | 0 |
| TCGA -CG- 5722- 11A-02R- 1602-13 | FALSE | FALSE | 1.68 | -0.57 | 0.91 | 0.56 | -0.17 | 1.37 | TRUE | 1.68 | -0.87 | -0.12 | 0 | 0.01 |
| TCGA -VQ-A928-01A-11R -A414- 31 | FALSE | FALSE | 1.68 | -0.86 | 0.27 | -0.58 | -0.4 | -1.14 | FALSE | -0.33 | 1.68 | 0.08 | 0.15 | 0.02 |
| TCGA -BR- 6456-01A- 11R-1802-13 | FALSE | FALSE | 1.66 | -0.05 | 0.14 | -0.01 | -0.07 | 0.02 | FALSE | 0.86 | 1.66 | 0.05 | 0.24 | -0.04 |
| TCGA -HU-A4GC -11A-11R -A251- 31 | TRUE | FALSE | 1.65 | -1.5 | 0.96 | -0.84 | -0.35 | -0.75 | FALSE | -0.17 | 1.65 | -0.03 | 0.23 | 0.05 |
| TCGA -HU-A4GH- 11A-11R-A36D-31 | TRUE | FALSE | 1.64 | -1.96 | 0.9 | -1.1 | -0.33 | -0.83 | FALSE | 0.22 | 1.64 | -0.04 | 0.27 | 0.03 |
| TCGA -HU-A4GY-11A-11R -A36D-31 | TRUE | FALSE | 1.63 | -1.66 | 0.89 | -0.92 | -0.29 | -0.87 | FALSE | 0.06 | 1.63 | -0.03 | 0.24 | 0.05 |
| TCGA -D7-A6EX-01A- 11R-A31P-31 | FALSE | FALSE | 1.62 | -0.87 | 0.3 | -1.06 | -0.73 | -0.88 | FALSE | -0.45 | 1.62 | 0.14 | 0.02 | 0.07 |
| TCGA -BR- 8058-01A- 31R-2343-13 | FALSE | FALSE | 1.61 | 1.16 | 0.22 | 1.08 | 0.16 | 1.27 | TRUE | 1.61 | -0.64 | -0.16 | 0.12 | -0.05 |
| TCGA -CG- 5733- 11A-01R- 1602-13 | FALSE | FALSE | 1.6 | -0.37 | 0.9 | 0.86 | -0.09 | 1.43 | TRUE | 1.6 | -1.65 | -0.15 | -0.12 | 0.02 |
| TCGA -CD- 8524-01A- 11R-2343-13 | FALSE | FALSE | 1.6 | 0.53 | 0.04 | 0.15 | -0.47 | 0.5 | FALSE | 0.25 | 1.6 | 0.06 | 0.18 | -0.01 |
| TCGA -MX -A5UJ -01A-11R -A31P- 31 | FALSE | FALSE | 1.59 | 0.03 | 0.96 | -0.3 | -0.1 | -0.59 | FALSE | 0.09 | 1.59 | 0.1 | 0.11 | 0.02 |
| TCGA -HU-A4HB- 11A- 11R-A251-31 | TRUE | FALSE | 1.58 | -1.27 | 0.93 | -0.83 | -0.05 | -0.66 | FALSE | -0.07 | 1.58 | -0.03 | 0.25 | 0.03 |
| TCGA -HU-A4GP-11A-21R -A251-31 | TRUE | FALSE | 1.58 | -1.32 | 0.91 | -0.82 | -0.21 | -0.27 | FALSE | 0.01 | 1.58 | -0.03 | 0.23 | 0.04 |
| TCGA -CG- 5720- 11A-01R- 1602-13 | FALSE | FALSE | 1.58 | -0.39 | 0.96 | 0.56 | -0.22 | 0.35 | TRUE | 1.58 | -1.81 | -0.13 | -0.17 | 0.02 |
| TCGA -CG- 5725-01A- 11R- 1602-13 | FALSE | FALSE | 1.56 | -0.6 | 0.14 | -0.65 | -0.5 | 2.46 | FALSE | -0.57 | 1.56 | 0.15 | -0.01 | 0.08 |
| TCGA -BR- 7715-11A-01R-2055-13 | FALSE | FALSE | 1.56 | 0.6 | 0.92 | 1.2 | 0.34 | 1.64 | TRUE | 1.56 | -1.94 | -0.18 | -0.12 | 0 |
| TCGA -D7-8572-01A-11R -2343- 13 | FALSE | FALSE | 1.56 | -0.04 | 0.14 | -0.06 | 0.55 | 0.43 | FALSE | 0.41 | 1.56 | 0.06 | 0.21 | -0.03 |
| TCGA -CG-4475-01A-01R- 1157-13 | FALSE | FALSE | 1.55 | -0.99 | 0.19 | -1.01 | -0.01 | -1.43 | FALSE | -1.09 | 1.55 | 0.05 | 0.21 | -0.03 |
| TCGA -IN-A7NT-01A-21R-A354- 31 | FALSE | FALSE | 1.55 | -0.8 | 0.25 | -0.59 | 0.5 | -0.89 | FALSE | -0.56 | 1.55 | 0.15 | 0.1 | -0.03 |
| TCGA -BR- 8366-01A- 11R-2343-13 | FALSE | FALSE | 1.54 | 3.08 | 0.37 | 1.98 | 2.39 | 1.98 | TRUE | 1.54 | -0.62 | -0.1 | 0.12 | -0.11 |
| TCGA -BR- 6564-01A- 12R-1884-13 | FALSE | FALSE | 1.53 | -0.09 | 0.29 | 0.31 | -0.28 | 0.28 | FALSE | 1.57 | 1.53 | -0.03 | 0.28 | -0.01 |
| TCGA -BR-4201-01A-01R-1131-13 | FALSE | FALSE | 1.53 | 0.8 | 0.94 | 0.19 | 1.23 | -0.75 | FALSE | -0.88 | 1.53 | 0.02 | 0.24 | -0.03 |
| TCGA -CD- 8529-01A- 11R-2343-13 | FALSE | FALSE | 1.52 | 0.96 | 0.12 | 0.8 | -0.05 | 0.39 | TRUE | 1.52 | -0.7 | -0.14 | 0.09 | -0.05 |
| TCGA -BR- 8080-01A- 11R-2343-13 | FALSE | FALSE | 1.51 | -0.22 | 0.1 | 0.14 | -0.01 | 0.25 | FALSE | 0.97 | 1.51 | -0.02 | 0.26 | 0 |
| TCGA -VQ-A8PD-01A-11R -A414-31 | FALSE | FALSE | 1.5 | 0.87 | 0.22 | 0.55 | 0.26 | 0.65 | TRUE | 1.5 | -1.73 | -0.17 | -0.04 | -0.04 |
| TCGA -VQ-A8DU-01A- 11R-A36D- 31 | FALSE | FALSE | 1.49 | -0.24 | 0.06 | -0.56 | -0.27 | -0.02 | FALSE | -0.68 | 1.49 | 0.14 | 0.03 | 0.05 |
| TCGA -FP-7916-01A-11R -2203-13 | FALSE | FALSE | 1.47 | 2.31 | 0.09 | 1.56 | 0.8 | 1.48 | TRUE | 1.47 | -1.33 | -0.15 | 0.04 | -0.09 |
| TCGA -CD- 8526-01A- 11R-2343-13 | FALSE | FALSE | 1.46 | 0.19 | 0.01 | -0.08 | -0.23 | -0.02 | FALSE | -0.17 | 1.46 | 0.16 | 0.02 | 0.02 |
| TCGA -RD-A8N9-01A-12R -A39E-31 | FALSE | FALSE | 1.46 | 0.01 | 0.05 | 0.27 | 0.12 | 0.33 | FALSE | 0.46 | 1.46 | -0.06 | 0.3 | 0 |
| TCGA -RD-A8N6-01A-11R -A36D-31 | FALSE | FALSE | 1.45 | -0.79 | 0.06 | -1.1 | -0.87 | -0.59 | FALSE | -0.32 | 1.45 | 0.08 | 0.09 | 0.04 |
| TCGA -BR- 8295-01A- 11R-2343-13 | TRUE | FALSE | 1.45 | -1.38 | 0.23 | -1.3 | -0.84 | -1 | FALSE | -1.14 | 1.45 | 0.15 | -0.06 | 0.11 |
| TCGA -D7-6524-01A-11R -1802- 13 | FALSE | FALSE | 1.43 | -0.72 | 0.3 | -0.34 | 0.39 | -0.81 | FALSE | 1.3 | 1.43 | -0.05 | 0.33 | -0.05 |
| TCGA -VQ-A94P-01A-13R-A414- 31 | FALSE | FALSE | 1.43 | -0.01 | 0.55 | 0.02 | -0.01 | 0.23 | FALSE | 0.32 | 1.43 | -0.05 | 0.28 | -0.01 |
| TCGA -BR- 8365-01A-21R-2343-13 | FALSE | FALSE | 1.42 | -0.3 | 0.37 | -0.06 | -0.12 | 0.36 | FALSE | 0.92 | 1.42 | -0.06 | 0.27 | 0.01 |
| TCGA -BR-A4J9-01A- 12R-A251-31 | FALSE | FALSE | 1.42 | -0.46 | 0.45 | -0.34 | -0.41 | -0.5 | FALSE | 0.34 | 1.42 | -0.04 | 0.22 | 0.04 |

| TCGA -D7-6525-01A-11R -1802- 13 | FALSE | FALSE | 1.4 | -0.3 | 0.17 | 0.02 | 0.54 | -0.42 | FALSE | 0.29 | 1.4 | 0.12 | 0.12 | -0.03 |
| --- | --- | --- | --- | --- | --- | --- | --- | --- | --- | --- | --- | --- | --- | --- |
| TCGA -BR- 7958-01A-21R-2343-13 | FALSE | FALSE | 1.39 | 3.32 | 0.31 | 2.25 | 1.54 | 2.7 | TRUE | 1.39 | -1.41 | -0.1 | 0.02 | -0.12 |
| TCGA -D7-6519-01A-11R -1802- 13 | FALSE | FALSE | 1.37 | -0.9 | 0.42 | -0.33 | -0.56 | 0.19 | FALSE | 0.03 | 1.37 | 0.14 | 0.04 | 0.02 |
| TCGA -BR- 6803-01A- 11R-1884-13 | FALSE | FALSE | 1.37 | -0.17 | 0.31 | 0.35 | -0.46 | 0.93 | FALSE | 1.53 | 1.37 | -0.05 | 0.22 | 0.04 |
| TCGA -FP-8209-01A-11R -2343-13 | FALSE | FALSE | 1.36 | -0.1 | 0.19 | 0.53 | 0.02 | 1.37 | TRUE | 1.36 | 0.46 | -0.14 | 0.21 | 0 |
| TCGA -RD-A8N2-01A-12R -A36D-31 | FALSE | FALSE | 1.36 | -0.84 | 0.56 | -0.32 | -0.26 | 0.31 | FALSE | 0.16 | 1.36 | -0.06 | 0.23 | 0.04 |
| TCGA -FP-8631-01A-11R -2402-13 | TRUE | FALSE | 1.34 | -1.16 | 0.12 | -1.05 | -0.54 | -1.34 | FALSE | -0.5 | 1.34 | 0.12 | 0.07 | 0.01 |
| TCGA -HU-A4GQ-01A-11R-A36D-31 | FALSE | FALSE | 1.32 | 1.4 | 0.98 | 0.37 | 0.83 | -0.31 | FALSE | -1.09 | 1.32 | 0.13 | 0.09 | -0.03 |
| TCGA -CG- 5721-01A- 11R- 1602-13 | FALSE | FALSE | 1.31 | 3.47 | 0.98 | 2.44 | 2.51 | 2.18 | TRUE | 1.31 | -1.34 | -0.05 | -0.06 | -0.1 |
| TCGA -KB-A93G-01A- 11R-A39E-31 | TRUE | FALSE | 1.31 | -1.03 | 0.06 | -0.59 | 1 | -1.26 | FALSE | 0.84 | 1.31 | -0.05 | 0.36 | -0.09 |
| TCGA -CG- 5724-01A- 11R- 1602-13 | FALSE | FALSE | 1.31 | 1.04 | 0.59 | 0.51 | 0.56 | -0.16 | FALSE | 0.5 | 1.31 | 0.18 | 0.04 | -0.02 |
| TCGA -CG- 5722-01A-21R- 1602-13 | FALSE | FALSE | 1.3 | 1.09 | 0.38 | 1.53 | 2.38 | 2.23 | TRUE | 1.3 | -1.04 | -0.05 | -0.12 | 0 |
| TCGA -HU-A4HD-01A-11R-A251- 31 | FALSE | FALSE | 1.28 | -0.98 | 0.06 | -0.8 | -0.56 | -0.09 | FALSE | -0.82 | 1.28 | 0.16 | -0.04 | 0.06 |
| TCGA -RD-A8N1-01A-12R -A36D-31 | FALSE | FALSE | 1.28 | 1.1 | 0.13 | 0.96 | 0.3 | 0.93 | TRUE | 1.28 | -1.46 | -0.14 | -0.06 | -0.02 |
| TCGA -BR- 7717-11A-01R-2055-13 | FALSE | FALSE | 1.27 | 0.55 | 0.96 | 1.12 | 0.31 | 1.55 | TRUE | 1.27 | -1.16 | -0.12 | -0.06 | -0.01 |
| TCGA -VQ-A94U-01A-12R -A414- 31 | TRUE | FALSE | 1.26 | -1.54 | 0.1 | -1.19 | -0.81 | -1.34 | FALSE | -0.44 | 1.26 | 0.08 | 0.06 | 0.06 |
| TCGA -VQ-A8PF-01A- 11R-A414-31 | FALSE | FALSE | 1.26 | 2.69 | 0.19 | 1.76 | 0.78 | 1.77 | TRUE | 1.26 | -2.22 | -0.16 | -0.09 | -0.07 |
| TCGA -CD- 8532-01A- 11R-2343-13 | FALSE | FALSE | 1.25 | 1.36 | 0.31 | 0.9 | 0.05 | 1.01 | TRUE | 1.25 | -1.5 | -0.13 | -0.05 | -0.04 |
| TCGA -BR- 7901-01A- 11R-2203-13 | FALSE | FALSE | 1.25 | 0.24 | 0.03 | 0.14 | -0.07 | 0.01 | FALSE | 0.61 | 1.25 | 0.01 | 0.24 | -0.05 |
| TCGA -BR- 8592-01A- 11R-2402-13 | TRUE | FALSE | 1.24 | -1.14 | 0.41 | -0.4 | -0.57 | -0.17 | FALSE | 0.86 | 1.24 | -0.08 | 0.25 | 0.03 |
| TCGA -VQ-A91Q-01A- 12R-A414-31 | FALSE | FALSE | 1.22 | -0.61 | 0.12 | -0.87 | -0.32 | -1.55 | FALSE | -0.13 | 1.22 | 0.09 | 0.13 | -0.03 |
| TCGA -CD- 8535-01A- 11R-2343-13 | TRUE | FALSE | 1.22 | -1.43 | 0.08 | -1.28 | -0.1 | -1.03 | FALSE | -0.85 | 1.22 | 0.17 | -0.03 | 0.03 |
| TCGA -BR- 8384-01A-21R-2402-13 | FALSE | FALSE | 1.21 | -0.35 | 0.31 | 0.15 | -0.23 | 0.87 | FALSE | 0.8 | 1.21 | -0.09 | 0.27 | 0.01 |
| TCGA -BR-A4J6-01A- 11R-A251-31 | TRUE | FALSE | 1.2 | -1.12 | 0.6 | -0.46 | -0.54 | -0.49 | FALSE | -0.33 | 1.2 | 0.02 | 0.1 | 0.07 |
| TCGA -BR- 6802-01A- 11R-1884-13 | FALSE | FALSE | 1.19 | 2.39 | 0.97 | 1.68 | 2.23 | 1.31 | TRUE | 1.19 | -1.02 | 0.02 | -0.06 | -0.13 |
| TCGA -BR-A4J8-01A- 11R-A251-31 | FALSE | FALSE | 1.19 | -0.69 | 0.11 | -0.58 | -0.45 | -0.7 | FALSE | 0.27 | 1.19 | 0.03 | 0.14 | 0.02 |
| TCGA -BR- 8369-01A- 11R-2343-13 | TRUE | FALSE | 1.19 | -1.47 | 0.2 | -1.15 | -0.81 | -0.98 | FALSE | -0.4 | 1.19 | 0.05 | 0.09 | 0.04 |
| TCGA -BR- 7723-01A- 11R-2055-13 | FALSE | FALSE | 1.18 | 0.57 | 0.02 | 0.29 | -0.05 | 1.12 | FALSE | -0.47 | 1.18 | 0.07 | 0.04 | 0.06 |
| TCGA -BR- 7196-01A- 11R-2055-13 | FALSE | FALSE | 1.18 | 1.33 | 0.1 | 1.05 | 0.05 | 1.14 | TRUE | 1.18 | 1.23 | -0.03 | 0.28 | -0.06 |
| TCGA -BR- 6801-01A- 11R-1884-13 | TRUE | FALSE | 1.18 | -1 | 0.23 | -0.73 | -0.2 | -0.91 | FALSE | 0.46 | 1.18 | 0.05 | 0.1 | 0.03 |
| TCGA -CG- 5721- 11A-01R- 1602-13 | FALSE | FALSE | 1.17 | -0.18 | 0.96 | 0.84 | 0.07 | 1.97 | TRUE | 1.17 | -1.71 | -0.13 | -0.17 | 0.03 |
| TCGA -BR- 6454-01A- 11R-1802-13 | FALSE | FALSE | 1.16 | 1.79 | 0.04 | 1.52 | 0.21 | 1.75 | TRUE | 1.16 | -1.15 | -0.03 | -0.08 | -0.07 |
| TCGA -D7-A6EY-01A-21R -A31P- 31 | FALSE | FALSE | 1.15 | 1.88 | 0.94 | 1.18 | 1.33 | 0.85 | TRUE | 1.15 | -1.1 | -0.03 | -0.06 | -0.07 |
| TCGA -BR- 8291-01A- 11R-2343-13 | FALSE | FALSE | 1.11 | -0.63 | 0.07 | 0 | -0.65 | 0.73 | TRUE | 1.11 | 1.19 | -0.15 | 0.33 | 0.02 |
| TCGA -HU-A4GN- 11A-12R-A251- 31 | TRUE | FALSE | 1.11 | -1.3 | 0.98 | -0.89 | -0.3 | -0.88 | FALSE | 0.25 | 1.11 | -0.06 | 0.22 | 0.01 |
| TCGA -BR- 8483-01A- 31R-2402-13 | FALSE | FALSE | 1.09 | -0.71 | 0.03 | -0.84 | -0.6 | -0.8 | FALSE | -1.09 | 1.09 | 0.13 | -0.03 | 0.05 |
| TCGA -HU-A4HB-01A- 12R-A251-31 | FALSE | FALSE | 1.09 | 0.72 | 0.56 | 0.65 | 0 | 0.85 | TRUE | 1.09 | -2.64 | -0.16 | -0.23 | -0.02 |

| TCGA -CG-4437-01A-01R- 1802-13 | FALSE | FALSE | 1.08 | 0.96 | 0.89 | 1.21 | 0.88 | 0.91 | TRUE | 1.08 | -0.21 | 0 | 0.01 | -0.05 |
| --- | --- | --- | --- | --- | --- | --- | --- | --- | --- | --- | --- | --- | --- | --- |
| TCGA -BR- 7716-01A-21R-2055-13 | FALSE | FALSE | 1.07 | 1.43 | 0.05 | 1.17 | 0.16 | 2.24 | TRUE | 1.07 | -0.32 | -0.02 | 0 | -0.03 |
| TCGA -BR- 8680-01A- 11R-2402-13 | TRUE | FALSE | 1.07 | -1.41 | 0.76 | -1.19 | -0.73 | -0.92 | FALSE | -1.54 | 1.07 | 0.18 | -0.12 | 0.09 |
| TCGA -BR- 8678-01A- 11R-2402-13 | FALSE | FALSE | 1.07 | 1.37 | 0.19 | -0.05 | 1.19 | 0.82 | FALSE | -0.62 | 1.07 | 0.18 | -0.01 | -0.01 |
| TCGA -D7-6520-01A-11R -1802- 13 | FALSE | FALSE | 1.06 | 0.19 | 0.07 | 0.06 | -0.52 | -0.97 | FALSE | 0.67 | 1.06 | 0 | 0.17 | 0 |
| TCGA -BR- 8371-01A- 11R-2343-13 | FALSE | FALSE | 1.06 | -0.77 | 0.62 | -0.38 | -0.41 | -0.3 | FALSE | 0.73 | 1.06 | -0.05 | 0.18 | 0.03 |
| TCGA -CG- 5734- 11A-01R- 1602-13 | FALSE | FALSE | 1.06 | -0.32 | 0.99 | 0.24 | 0.15 | 0.09 | TRUE | 1.06 | -1.38 | -0.12 | -0.08 | -0.01 |
| TCGA -F1- 6875-01A- 11R-2055-13 | TRUE | FALSE | 1.06 | -2.19 | 0.05 | -1.58 | 0.31 | 0.34 | FALSE | -0.41 | 1.06 | 0.15 | -0.05 | 0.05 |
| TCGA -BR- 8081-01A- 11R-2343-13 | FALSE | FALSE | 1.06 | 2.37 | 0.98 | 1.47 | 1.52 | 0.81 | TRUE | 1.06 | -0.95 | -0.09 | 0.05 | -0.1 |
| TCGA -RD-A7BW-01A- 11R-A32D- 31 | FALSE | FALSE | 1.05 | 1.2 | 0.2 | 0.86 | 0.18 | 2.08 | FALSE | 0.91 | 1.05 | -0.08 | 0.26 | -0.02 |
| TCGA -B7-A5TI-01A-11R -A31P- 31 | FALSE | FALSE | 1.05 | -0.07 | 0.68 | -0.27 | -0.44 | -0.47 | FALSE | -0.33 | 1.05 | 0.02 | 0.08 | 0.04 |
| TCGA -D7-8570-01A-11R -2343- 13 | FALSE | FALSE | 1.04 | 2.21 | 0.08 | 1.67 | 1.22 | 0.72 | TRUE | 1.04 | -1.52 | -0.19 | 0 | -0.03 |
| TCGA -CG- 5732-01A- 11R- 1602-13 | FALSE | FALSE | 1.04 | -0.15 | 0.52 | 0.45 | -0.25 | 0.75 | TRUE | 1.04 | -1.71 | -0.08 | -0.19 | 0 |
| TCGA -HF-7132-01A-11R-2055- 13 | FALSE | FALSE | 1.04 | 0.57 | 0.95 | 0.59 | 0.31 | 0.61 | TRUE | 1.04 | -1.39 | -0.12 | -0.07 | -0.01 |
| TCGA -VQ-A923-01A-11R -A414- 31 | FALSE | FALSE | 1.04 | 2.18 | 0.07 | 1.25 | 0.74 | 1.84 | TRUE | 1.04 | -2.38 | -0.11 | -0.17 | -0.07 |
| TCGA -BR- 6453-11A-01R-1802-13 | FALSE | FALSE | 1.03 | -0.68 | 0.97 | 0.17 | 0 | 0.37 | TRUE | 1.03 | -1.43 | -0.13 | -0.1 | 0.01 |
| TCGA -RD-A8MV-01A-11R-A36D-31 | FALSE | FALSE | 1.03 | 1 | 0.26 | 0.96 | 0.5 | 0.82 | TRUE | 1.03 | -2.76 | -0.11 | -0.2 | -0.11 |
| TCGA -BR- 7197-01A- 11R-2203-13 | TRUE | FALSE | 1.02 | -1.63 | 0.05 | -1.27 | -0.84 | -1.17 | FALSE | -0.39 | 1.02 | 0.08 | 0 | 0.07 |
| TCGA -3M -AB47-01A-22R -A414- 31 | FALSE | FALSE | 1.02 | -0.79 | 0.66 | -0.19 | -0.04 | -0.3 | FALSE | 0.21 | 1.02 | -0.04 | 0.16 | 0.04 |
| TCGA -BR- 8367-01A- 11R-2343-13 | FALSE | FALSE | 1 | -1.01 | 0.46 | -0.65 | -0.57 | -0.46 | FALSE | 0.08 | 1 | -0.06 | 0.15 | 0.07 |
| TCGA -CD-A4MG-01A-11R -A251-31 | FALSE | FALSE | 0.98 | -0.65 | 0.95 | -0.53 | -0.09 | -0.92 | FALSE | -0.8 | 0.98 | 0.06 | 0.01 | 0.06 |
| TCGA -BR- 8677-01A- 11R-2402-13 | FALSE | FALSE | 0.97 | 2.26 | 0.18 | 1.28 | 0.8 | 1.7 | TRUE | 0.97 | 0.64 | 0.01 | 0.18 | -0.09 |
| TCGA -CD- 5801-01A- 11R- 1602-13 | FALSE | FALSE | 0.97 | 2.65 | 0.34 | 2.14 | 0.46 | 2.94 | TRUE | 0.97 | -1.05 | -0.03 | -0.11 | -0.04 |
| TCGA -IP- 7968-01A- 11R-2203-13 | FALSE | FALSE | 0.97 | 0.9 | 0.05 | 0.46 | 0.24 | 0.73 | FALSE | 0.09 | 0.97 | 0.1 | 0.08 | -0.03 |
| TCGA -BR- 7716-11A-01R-2055-13 | FALSE | FALSE | 0.96 | -0.26 | 0.95 | 0.43 | -0.06 | 1.16 | TRUE | 0.96 | -0.71 | -0.09 | -0.01 | 0.01 |
| TCGA -BR-A44U-01A- 11R-A36D- 31 | FALSE | FALSE | 0.95 | -0.69 | 0.35 | -0.77 | -0.49 | -1.01 | FALSE | -0.94 | 0.95 | 0.08 | 0.02 | 0.04 |
| TCGA -D7-A747-01A-22R -A33Y-31 | FALSE | FALSE | 0.93 | 0.04 | 0.31 | 0.05 | -0.2 | 0.25 | FALSE | 0.66 | 0.93 | -0.08 | 0.19 | 0.03 |
| TCGA -CG-4442-01A-01R- 1157-13 | FALSE | FALSE | 0.93 | 0.34 | 1 | -0.37 | 0.55 | -0.72 | FALSE | -1.9 | 0.93 | 0.13 | -0.03 | 0.02 |
| TCGA -RD-A8N5-01A-12R -A36D-31 | FALSE | FALSE | 0.92 | -1.65 | 0.14 | -0.84 | -0.13 | -1.21 | FALSE | 0.73 | 0.92 | -0.07 | 0.28 | -0.06 |
| TCGA -BR- 8286-01A- 12R-2343-13 | FALSE | FALSE | 0.91 | 0.39 | 0.32 | 0.09 | -0.24 | 0.19 | FALSE | -0.71 | 0.91 | 0.09 | -0.03 | 0.08 |
| TCGA -VQ-A94T-01A-11R -A414-31 | FALSE | FALSE | 0.91 | -2.02 | 0.42 | -1.1 | 1.08 | -1.11 | FALSE | -1.58 | 0.91 | 0.16 | -0.07 | 0.04 |
| TCGA -CD- 5799-01A- 11R- 1602-13 | FALSE | FALSE | 0.9 | -1.15 | 0.72 | -0.67 | -0.42 | -0.95 | FALSE | -0.22 | 0.9 | 0.1 | 0.01 | 0.03 |
| TCGA -RD-A8N4-01A-21R -A36D-31 | FALSE | FALSE | 0.89 | 0.36 | 0.73 | 0.06 | -0.01 | 0.5 | FALSE | 0.37 | 0.89 | -0.05 | 0.18 | 0.01 |
| TCGA -BR- 6709-01A- 11R-1884-13 | FALSE | FALSE | 0.88 | 2.04 | 0.27 | 2.06 | 1.1 | 2.31 | TRUE | 0.88 | 0.65 | -0.01 | 0.17 | -0.06 |
| TCGA -BR- 8679-01A- 11R-2402-13 | FALSE | FALSE | 0.86 | -0.6 | 0.16 | -0.95 | -0.18 | -1.34 | FALSE | -1.16 | 0.86 | 0.1 | 0.04 | 0 |
| TCGA -D7-5577-01A-01R -1602- 13 | FALSE | FALSE | 0.86 | 2.97 | 0.47 | 1.94 | 1.41 | 1.83 | TRUE | 0.86 | -1.4 | -0.05 | -0.11 | -0.06 |
| TCGA -BR- 8590-01A- 11R-2402-13 | FALSE | FALSE | 0.86 | -0.15 | 0.24 | 0.09 | -0.27 | 0.28 | FALSE | 0.88 | 0.86 | -0.09 | 0.22 | 0 |

| TCGA -R5-A7ZF-01A- 11R-A354-31 | FALSE | FALSE | 0.84 | 0.23 | 0.53 | -0.55 | -0.1 | -0.77 | FALSE | -1.03 | 0.84 | 0.22 | -0.13 | 0.02 |
| --- | --- | --- | --- | --- | --- | --- | --- | --- | --- | --- | --- | --- | --- | --- |
| TCGA -BR- 8686-01A- 11R-2402-13 | FALSE | FALSE | 0.82 | 0.96 | 0.1 | 0.92 | -0.25 | 1.52 | TRUE | 0.82 | -0.73 | -0.09 | 0.02 | -0.04 |
| TCGA -BR- 7704-11A-01R-2055-13 | FALSE | FALSE | 0.8 | -1.1 | 0.9 | -0.15 | -0.19 | 0.48 | FALSE | 1.12 | 0.8 | -0.08 | 0.22 | -0.01 |
| TCGA -BR- 7703-11A-01R-2055-13 | FALSE | FALSE | 0.8 | -0.31 | 0.95 | 0.42 | -0.23 | 0.68 | TRUE | 0.8 | -1.46 | -0.12 | -0.12 | 0.02 |
| TCGA -CD- 5800-01A- 11R- 1602-13 | FALSE | FALSE | 0.79 | -1.05 | 0.08 | -0.68 | -0.96 | -0.01 | FALSE | 0.15 | 0.79 | 0.13 | -0.07 | 0.03 |
| TCGA -BR- 8060-01A- 11R-2343-13 | FALSE | FALSE | 0.79 | 0.41 | 0.16 | 0.03 | 0.49 | -0.46 | FALSE | 0.13 | 0.79 | 0.05 | 0.09 | -0.02 |
| TCGA -BR- 8687-01A- 11R-2402-13 | FALSE | FALSE | 0.79 | -0.89 | 0.38 | -0.78 | -0.69 | -1.01 | FALSE | -0.27 | 0.79 | 0.03 | 0.04 | 0.05 |
| TCGA -BR- 6458-01A- 11R-1802-13 | FALSE | FALSE | 0.79 | 1.02 | 0.07 | 0.82 | 0.12 | 0.64 | TRUE | 0.79 | 0.88 | 0.01 | 0.15 | -0.02 |
| TCGA -HU-8608-01A-11R -2402- 13 | FALSE | FALSE | 0.76 | 3.81 | 0.47 | 2.35 | 6.15 | 2 | TRUE | 0.76 | -2.61 | -0.12 | -0.15 | -0.12 |
| TCGA -BR- 7704-01A- 11R-2055-13 | FALSE | FALSE | 0.75 | 2.22 | 0.19 | 1.59 | 0.36 | 1.24 | TRUE | 0.75 | -0.41 | -0.01 | -0.08 | 0.02 |
| TCGA -VQ-A91Y-01A- 11R-A414-31 | FALSE | FALSE | 0.75 | 1.13 | 0.05 | 0.5 | 0.08 | 0.11 | FALSE | 0.68 | 0.75 | -0.07 | 0.24 | -0.04 |
| TCGA -B7-A5TK-01A- 12R-A36D- 31 | FALSE | FALSE | 0.73 | 4.37 | 0.53 | 2.56 | 3.54 | 2.84 | TRUE | 0.73 | -0.41 | -0.04 | 0.11 | -0.13 |
| TCGA -BR-A4J7-01A- 31R-A251-31 | FALSE | FALSE | 0.73 | 1.41 | 0.26 | 0.87 | 0.25 | 2.02 | TRUE | 0.73 | 0.68 | -0.08 | 0.21 | -0.02 |
| TCGA -BR- 7707-01A- 11R-2055-13 | FALSE | FALSE | 0.72 | 1.46 | 1 | 0.9 | 1.41 | -0.06 | FALSE | -0.12 | 0.72 | 0.2 | -0.06 | -0.04 |
| TCGA -FP-A4BF-01A-12R -A36D-31 | FALSE | FALSE | 0.72 | 2.24 | 0.22 | 1.49 | 0.5 | 1.52 | TRUE | 0.72 | -0.27 | -0.07 | 0.13 | -0.1 |
| TCGA -FP-7829-01A-11R -2055-13 | FALSE | FALSE | 0.71 | -1.49 | 0.66 | -1.19 | -0.65 | -1.52 | FALSE | -0.57 | 0.71 | 0.06 | 0.01 | 0.04 |
| TCGA -BR-4370-01A-01R-1157-13 | FALSE | FALSE | 0.7 | 1.09 | 0.95 | 0.28 | 1.08 | -0.19 | FALSE | -0.11 | 0.7 | -0.05 | 0.24 | -0.08 |
| TCGA -CD- 8533-01A- 11R-2343-13 | FALSE | FALSE | 0.69 | -1.81 | 0.01 | -1.31 | -1.11 | -1.39 | FALSE | -0.3 | 0.69 | 0.08 | -0.05 | 0.07 |
| TCGA -VQ-A94R-01A- 11R-A414-31 | FALSE | FALSE | 0.68 | 0.57 | 0.14 | 0.04 | -0.21 | -0.01 | FALSE | 0.08 | 0.68 | 0.02 | 0.04 | 0.04 |
| TCGA -VQ-A8DV-01A- 12R-A36D- 31 | FALSE | FALSE | 0.68 | -1.07 | 0.03 | -1.14 | -0.47 | -0.36 | FALSE | -1.16 | 0.68 | 0.09 | -0.08 | 0.08 |
| TCGA -3M -AB46-01A-11R -A414- 31 | FALSE | FALSE | 0.67 | -0.09 | 0.11 | -0.77 | -0.41 | -0.97 | FALSE | -1.51 | 0.67 | 0.1 | -0.04 | 0.03 |
| TCGA -D7-6818-01A-11R -1884- 13 | FALSE | FALSE | 0.67 | -0.87 | 0.1 | -0.25 | -0.3 | -1.18 | FALSE | 1.05 | 0.67 | -0.03 | 0.14 | 0 |
| TCGA -BR- 8381-01A- 11R-2402-13 | FALSE | FALSE | 0.67 | 2.4 | 0.26 | 1.48 | 0.42 | 1.83 | TRUE | 0.67 | -0.32 | -0.06 | 0.04 | -0.04 |
| TCGA -CG-4440-01A-01R- 1157-13 | FALSE | FALSE | 0.67 | -0.08 | 0.55 | -0.65 | -0.12 | -0.57 | FALSE | -1.29 | 0.67 | 0.14 | -0.08 | 0.02 |
| TCGA -HU-8604-01A-11R -2402- 13 | FALSE | FALSE | 0.65 | 1.53 | 0.44 | 1.36 | 5.55 | 1.41 | TRUE | 0.65 | -0.71 | -0.01 | -0.02 | -0.08 |
| TCGA -CG- 5717-01A- 11R- 1602-13 | FALSE | FALSE | 0.65 | 0.42 | 0.84 | 0.23 | 1.08 | 0.37 | TRUE | 0.65 | -0.95 | -0.07 | -0.05 | -0.03 |
| TCGA -BR- 7851-11A-01R-2203-13 | FALSE | FALSE | 0.64 | -0.27 | 0.99 | 0.02 | 0.58 | 0.35 | TRUE | 0.64 | -1.71 | -0.13 | -0.13 | 0.01 |
| TCGA -BR- 8284-01A- 11R-2343-13 | FALSE | FALSE | 0.63 | 0.96 | 0.8 | 0.87 | 0.05 | 2.06 | TRUE | 0.63 | -0.19 | -0.08 | 0.04 | 0.01 |
| TCGA -IN-AB1X-01A- 11R-A39E- 31 | FALSE | FALSE | 0.63 | 1.09 | 0.78 | 1.03 | 1.48 | 1.55 | TRUE | 0.63 | -1.88 | -0.03 | -0.15 | -0.11 |
| TCGA -BR- 6710-01A- 11R-1884-13 | FALSE | FALSE | 0.62 | -1.06 | 0.95 | -0.36 | -0.22 | 0.28 | TRUE | 0.62 | -1.02 | -0.11 | -0.04 | 0 |
| TCGA -CG-4462-01A-01R- 1157-13 | FALSE | FALSE | 0.61 | -0.11 | 0.22 | -0.02 | 0.36 | -0.68 | FALSE | 0.71 | 0.61 | -0.18 | 0.34 | -0.04 |
| TCGA -VQ-A8P5-01A-11R-A39E-31 | FALSE | FALSE | 0.61 | 0.67 | 0.07 | 0.2 | 0.03 | -0.34 | FALSE | -0.58 | 0.61 | 0.1 | 0.01 | -0.02 |
| TCGA -CG-4441-01A-01R- 1802-13 | FALSE | FALSE | 0.61 | 0.22 | 0.74 | 0.05 | 0.17 | -0.27 | FALSE | 0.19 | 0.61 | 0.02 | 0.11 | -0.03 |
| TCGA -VQ-A8PB-01A-11R -A39E-31 | FALSE | FALSE | 0.59 | -0.23 | 1 | -0.29 | 0.05 | 0.06 | FALSE | -1.78 | 0.59 | 0.12 | -0.06 | 0.03 |
| TCGA -D7-A6F0-01A-11R-A31P-31 | FALSE | FALSE | 0.59 | 1.19 | 0.05 | 0.21 | -0.1 | 0.44 | FALSE | -0.56 | 0.59 | 0.07 | -0.05 | 0.05 |
| TCGA -D7-A4YU-01A-21R-A251-31 | FALSE | FALSE | 0.57 | 2.24 | 0.32 | 1.25 | 0.83 | 0.88 | TRUE | 0.57 | -1.16 | -0.04 | -0.08 | -0.06 |
| TCGA -BR- 6565-01A- 11R-1802-13 | FALSE | FALSE | 0.54 | 1.44 | 0.2 | 0.95 | 0.44 | 0.64 | TRUE | 0.54 | 0.27 | 0.04 | 0.02 | -0.02 |

| TCGA -CG-4443-01A-01R- 1157-13 | FALSE | FALSE | 0.54 | -1.48 | 0.32 | -1.29 | -0.76 | -1.38 | FALSE | -1.05 | 0.54 | 0.03 | -0.07 | 0.11 |
| --- | --- | --- | --- | --- | --- | --- | --- | --- | --- | --- | --- | --- | --- | --- |
| TCGA -VQ-A94O-01A-11R-A414- 31 | FALSE | FALSE | 0.52 | -0.64 | 0.02 | -0.47 | -0.26 | -0.7 | FALSE | -0.65 | 0.52 | 0.13 | -0.09 | 0.03 |
| TCGA -IN-8663-01A-11R -2402-13 | FALSE | FALSE | 0.52 | -0.7 | 0.15 | -0.38 | 2.88 | -0.85 | FALSE | -0.29 | 0.52 | 0.13 | 0 | -0.04 |
| TCGA -HU-8238-11A-01R -2343- 13 | FALSE | FALSE | 0.52 | -0.72 | 1 | -0.18 | 0.36 | 0.87 | TRUE | 0.52 | -1.82 | -0.13 | -0.16 | 0.02 |
| TCGA -BR-4369-01A-01R-1157-13 | FALSE | FALSE | 0.51 | 0.3 | 0.02 | -0.11 | -0.28 | -0.58 | FALSE | -0.56 | 0.51 | 0 | 0.02 | 0.05 |
| TCGA -D7-6822-01A-11R -1884- 13 | FALSE | FALSE | 0.51 | 0.75 | 0.04 | -0.08 | 0.49 | 1.56 | FALSE | -0.47 | 0.51 | 0.07 | 0 | 0 |
| TCGA -VQ-A8DZ -01A-11R -A36D-31 | FALSE | FALSE | 0.51 | -0.71 | 0.14 | -0.61 | -0.51 | 0.61 | FALSE | -0.4 | 0.51 | 0.11 | -0.09 | 0.05 |
| TCGA -BR- 8373-01A- 11R-2343-13 | FALSE | FALSE | 0.51 | -0.4 | 0.02 | -0.71 | -0.39 | -0.71 | FALSE | 0.24 | 0.51 | -0.03 | 0.11 | 0 |
| TCGA -BR- 8382-01A- 11R-2402-13 | FALSE | FALSE | 0.5 | 2.32 | 0.98 | 1 | 0.95 | -0.63 | FALSE | 0.35 | 0.5 | 0.06 | 0.12 | -0.1 |
| TCGA -FP-8210-01A-11R -2343-13 | FALSE | FALSE | 0.49 | 0.27 | 0.15 | 0.52 | -0.1 | 1.26 | FALSE | 1.85 | 0.49 | -0.15 | 0.23 | 0 |
| TCGA -R5-A7O7 -01A-11R -A33Y-31 | FALSE | FALSE | 0.49 | 0 | 0.19 | -0.18 | 0.45 | -0.32 | FALSE | -0.21 | 0.49 | 0.11 | -0.06 | 0.02 |
| TCGA -CD-A486-01A- 11R-A24K- 31 | FALSE | FALSE | 0.49 | -0.23 | 0.12 | -0.31 | -0.44 | -0.88 | FALSE | -0.06 | 0.49 | 0.04 | 0.05 | -0.02 |
| TCGA -IN-A7NU-01A-22R-A354-31 | FALSE | FALSE | 0.48 | 0.55 | 0.28 | 0.5 | 0.35 | -0.25 | FALSE | 0.62 | 0.48 | 0.04 | 0.05 | -0.03 |
| TCGA -CD- 8528-01A- 11R-2343-13 | FALSE | FALSE | 0.47 | -0.79 | 0.85 | -0.65 | 0.1 | -0.75 | FALSE | -1.16 | 0.47 | 0.08 | -0.04 | 0.03 |
| TCGA -BR- 8683-01A- 11R-2402-13 | FALSE | FALSE | 0.46 | 0.27 | 0.03 | 0.03 | 0.34 | 0.26 | FALSE | 0.55 | 0.46 | 0.01 | 0.12 | -0.05 |
| TCGA -CD- 8527-01A- 11R-2343-13 | FALSE | FALSE | 0.45 | -0.11 | 0.2 | 0.05 | -0.49 | 0.51 | FALSE | -0.2 | 0.45 | 0.1 | -0.03 | -0.01 |
| TCGA -VQ-A91Z-01A- 11R-A414-31 | FALSE | FALSE | 0.45 | -1.64 | 0.07 | -1.48 | -0.95 | -0.35 | FALSE | -0.7 | 0.45 | 0.13 | -0.12 | 0.06 |
| TCGA -R5-A7ZI -01A-11R -A354- 31 | FALSE | FALSE | 0.44 | 1.67 | 0.98 | 1.69 | 2.22 | 2.08 | TRUE | 0.44 | -1.99 | -0.05 | -0.2 | -0.06 |
| TCGA -D7-6527-01A-11R -1802- 13 | FALSE | FALSE | 0.42 | -0.34 | 0.32 | -0.22 | -0.25 | -0.5 | FALSE | 0.23 | 0.42 | 0.03 | -0.03 | 0.06 |
| TCGA -CG-4460-01A-01R- 1157-13 | FALSE | FALSE | 0.42 | 0.28 | 0.8 | -0.3 | 0.37 | -0.11 | FALSE | -0.97 | 0.42 | 0.08 | -0.06 | 0.03 |
| TCGA -RD-A8MW-01A- 11R-A36D- 31 | FALSE | FALSE | 0.4 | 1.63 | 0.14 | 0.91 | 0.58 | 0.58 | FALSE | 0.22 | 0.4 | -0.03 | 0.17 | -0.08 |
| TCGA -D7-8579-01A-11R -2343- 13 | FALSE | FALSE | 0.4 | -1.28 | 0.27 | -0.65 | -0.38 | -0.57 | FALSE | 1.09 | 0.4 | -0.12 | 0.18 | 0.01 |
| TCGA -B7-5818-01A-11R- 1602- 13 | FALSE | FALSE | 0.39 | 1.81 | 0.66 | 1.32 | 0.53 | 0.95 | TRUE | 0.39 | -1.03 | -0.04 | -0.07 | -0.05 |
| TCGA -BR-4187-01A-01R-1131-13 | FALSE | FALSE | 0.39 | -0.16 | 0.6 | 0.16 | -0.36 | 0.55 | TRUE | 0.39 | 1.1 | -0.13 | 0.32 | -0.01 |
| TCGA -HF-7133-01A-11R-2055- 13 | FALSE | FALSE | 0.39 | 0.77 | 0.19 | 0.52 | 0.76 | 0.32 | TRUE | 0.39 | -0.23 | 0.01 | -0.01 | -0.04 |
| TCGA -BR- 6566-01A- 11R-1802-13 | FALSE | FALSE | 0.38 | 0.84 | 0.99 | 0.92 | 0.55 | 0.05 | FALSE | 0.95 | 0.38 | 0.01 | 0.08 | -0.04 |
| TCGA -VQ-A8P2-01A-11R-A36D-31 | FALSE | FALSE | 0.36 | -1.32 | 1 | -0.92 | 0.07 | -1 | FALSE | -1.53 | 0.36 | 0.14 | -0.19 | 0.09 |
| TCGA -MX -A666-01A-11R-A31P-31 | FALSE | FALSE | 0.35 | -1.63 | 0.03 | -0.93 | -0.65 | -0.05 | FALSE | -0.2 | 0.35 | 0.13 | -0.18 | 0.09 |
| TCGA -D7-6526-01A-11R -1802- 13 | FALSE | FALSE | 0.34 | -1.43 | 0.05 | -1.07 | -0.72 | -1.1 | FALSE | -0.38 | 0.34 | 0.11 | -0.1 | 0.03 |
| TCGA -D7-6815-01A-11R -1884- 13 | FALSE | FALSE | 0.33 | -0.42 | 0.25 | -0.41 | 0.35 | -0.52 | FALSE | -0.09 | 0.33 | 0.01 | 0 | 0.03 |
| TCGA -D7-A748-01A-12R -A32D-31 | FALSE | FALSE | 0.33 | 0.62 | 0.34 | 0.43 | 0.43 | -0.39 | FALSE | 0.93 | 0.33 | -0.16 | 0.25 | -0.03 |
| TCGA -FP-8099-01A-11R -2343-13 | FALSE | FALSE | 0.33 | 0.21 | 0.19 | -0.25 | -0.39 | -1.35 | FALSE | 0.16 | 0.33 | 0.06 | 0.03 | -0.04 |
| TCGA -VQ-A8PK-01A-12R -A414-31 | FALSE | FALSE | 0.33 | 0.25 | 0.12 | -0.1 | 0.26 | -0.24 | FALSE | -0.31 | 0.33 | 0.09 | 0.02 | -0.06 |
| TCGA -CD- 8525-01A- 11R-2343-13 | FALSE | FALSE | 0.32 | 1.07 | 0.05 | 0.82 | -0.31 | 1.15 | TRUE | 0.32 | 0.37 | 0.09 | -0.01 | -0.04 |
| TCGA -BR-4256-01A-01R-1131-13 | FALSE | FALSE | 0.32 | 2.3 | 0.96 | 1.39 | 1.49 | 0.86 | TRUE | 0.32 | 0.11 | -0.08 | 0.23 | -0.12 |
| TCGA -D7-A6EV-01A- 11R-A31P-31 | FALSE | FALSE | 0.31 | -0.98 | 0.85 | -0.94 | -0.64 | -0.97 | FALSE | -1.34 | 0.31 | 0.15 | -0.16 | 0.05 |
| TCGA -HJ-7597-01A-21R -2203-13 | FALSE | FALSE | 0.3 | 1.8 | 0.96 | 0.71 | 0.54 | 0.99 | TRUE | 0.3 | -0.38 | 0.12 | -0.09 | -0.1 |

| TCGA -BR- 8690-01A- 11R-2402-13 | FALSE | FALSE | 0.3 | 2.5 | 0.14 | 1.33 | 0.24 | 1.45 | TRUE | 0.3 | -0.4 | 0 | 0.01 | -0.07 |
| --- | --- | --- | --- | --- | --- | --- | --- | --- | --- | --- | --- | --- | --- | --- |
| TCGA -VQ-A8PH-01A-12R -A414-31 | FALSE | FALSE | 0.29 | -0.93 | 0.05 | -0.34 | -0.43 | 0.89 | FALSE | -0.71 | 0.29 | 0.14 | -0.17 | 0.05 |
| TCGA -CD- 5804-01A- 12R-2055-13 | FALSE | FALSE | 0.29 | 0.03 | 0.11 | 0.2 | -0.4 | 0.05 | FALSE | 0.98 | 0.29 | -0.03 | 0.09 | -0.02 |
| TCGA -BR- 8588-01A- 11R-2402-13 | FALSE | FALSE | 0.29 | 0.03 | 0.72 | 0.15 | 0.15 | 0.81 | TRUE | 0.29 | 0.37 | -0.06 | 0.12 | -0.01 |
| TCGA -VQ-A8PO-01A- 11R-A414-31 | FALSE | FALSE | 0.27 | 1.53 | 0.99 | 0.98 | 0.53 | 0.99 | TRUE | 0.27 | -1.18 | 0.01 | -0.14 | -0.07 |
| TCGA -CG-4466-01A-01R- 1157-13 | FALSE | FALSE | 0.27 | 0.03 | 0.38 | -0.92 | -0.27 | -1.19 | FALSE | -1.94 | 0.27 | 0.14 | -0.15 | 0.03 |
| TCGA -BR-A4CR-01A- 11R-A24K- 31 | FALSE | FALSE | 0.25 | -1.95 | 0.13 | -1.52 | -0.42 | -1.38 | FALSE | -1.07 | 0.25 | 0.08 | -0.1 | 0.05 |
| TCGA -D7-5578-01A-01R -1602- 13 | FALSE | FALSE | 0.25 | 0.17 | 0.38 | 0.35 | 0.02 | 0.13 | FALSE | 0.99 | 0.25 | 0.04 | 0 | 0 |
| TCGA -IN-8462-01A-11R -2343-13 | FALSE | FALSE | 0.24 | -0.26 | 0.46 | -0.37 | 0.01 | -0.71 | FALSE | 0.57 | 0.24 | 0 | 0.06 | -0.02 |
| TCGA -BR-A4CS-01A- 11R-A24K- 31 | FALSE | FALSE | 0.24 | -1.05 | 0.05 | -0.73 | -0.08 | -1.09 | FALSE | -0.26 | 0.24 | 0.02 | 0.02 | 0 |
| TCGA -HU-A4H3-01A-21R-A251-31 | FALSE | FALSE | 0.24 | -0.07 | 1 | -0.03 | -0.08 | 0.15 | FALSE | -0.83 | 0.24 | 0.13 | -0.08 | -0.03 |
| TCGA -CD-A4MH-01A-11R -A251-31 | FALSE | FALSE | 0.24 | -0.59 | 0.06 | -0.51 | -0.01 | -0.08 | FALSE | -0.45 | 0.24 | 0.06 | -0.06 | 0.03 |
| TCGA -BR- 6707-01A- 11R-1884-13 | FALSE | FALSE | 0.21 | 2.26 | 0.67 | 1.54 | 0.81 | 2.1 | TRUE | 0.21 | -0.77 | 0 | -0.12 | 0 |
| TCGA -IP- 7968- 11A-01R-2203-13 | FALSE | FALSE | 0.21 | -1.35 | 0.93 | -0.9 | -0.27 | -0.74 | FALSE | 0.38 | 0.21 | -0.02 | 0.05 | 0 |
| TCGA -RD-A7C1-01A-11R -A32D-31 | FALSE | FALSE | 0.21 | 1.75 | 0.69 | 1.18 | 1.13 | 1.3 | TRUE | 0.21 | -1.49 | -0.02 | -0.19 | -0.03 |
| TCGA -D7-6521-01A-11R -1802- 13 | FALSE | FALSE | 0.2 | -0.14 | 0.09 | 0.38 | -0.21 | 0.94 | FALSE | 1.21 | 0.2 | -0.12 | 0.15 | 0 |
| TCGA -B7-A5TJ-01A-11R -A31P- 31 | FALSE | FALSE | 0.2 | -1.12 | 0.47 | -0.92 | -0.61 | -1.14 | FALSE | -0.46 | 0.2 | 0.05 | -0.11 | 0.08 |
| TCGA -MX -A5UG-01A-21R -A31P- 31 | FALSE | FALSE | 0.2 | -0.18 | 0.11 | 0.27 | 0.15 | 0.29 | FALSE | 1.74 | 0.2 | -0.16 | 0.24 | -0.03 |
| TCGA -VQ-A8PJ -01A-11R -A414- 31 | FALSE | FALSE | 0.19 | -1.07 | 0.07 | -1.03 | -0.71 | -0.09 | FALSE | -0.66 | 0.19 | 0.12 | -0.13 | 0.03 |
| TCGA -BR- 8060-11A-01R-2343-13 | FALSE | FALSE | 0.18 | -1.32 | 0.92 | -0.4 | -0.21 | 0.78 | FALSE | 0.68 | 0.18 | -0.1 | 0.12 | 0.01 |
| TCGA -HU-A4H5-01A-21R-A251-31 | FALSE | FALSE | 0.17 | -0.14 | 0.11 | -0.57 | -0.48 | -0.81 | FALSE | -0.93 | 0.17 | 0.09 | -0.14 | 0.06 |
| TCGA -CG- 5723-01A- 11R- 1602-13 | FALSE | FALSE | 0.17 | 2.8 | 0.99 | 1.69 | 1.63 | 1.43 | TRUE | 0.17 | -0.09 | 0.05 | 0.03 | -0.1 |
| TCGA -IN-AB1V-11A- 11R-A414-31 | FALSE | FALSE | 0.16 | -1.78 | 0.94 | -1.17 | 0.16 | -0.41 | FALSE | -0.11 | 0.16 | -0.03 | 0.02 | 0.04 |
| TCGA -BR- 8484-01A- 11R-2402-13 | FALSE | FALSE | 0.13 | 1.26 | 0.18 | 0.74 | 0.45 | 0.58 | TRUE | 0.13 | -1.58 | -0.14 | -0.05 | -0.04 |
| TCGA -VQ-A8PE-01A- 11R-A414-31 | FALSE | FALSE | 0.13 | 0.06 | 0.09 | -0.01 | 0.19 | 0.4 | FALSE | -0.49 | 0.13 | 0.1 | -0.1 | 0 |
| TCGA -VQ-AA6D-01A- 11R-A414-31 | FALSE | FALSE | 0.12 | -0.9 | 0.75 | -1.05 | 0.31 | -0.28 | FALSE | -1.27 | 0.12 | 0.14 | -0.11 | -0.02 |
| TCGA -VQ-A92D-01A- 11R-A414-31 | FALSE | FALSE | 0.12 | -0.37 | 0.36 | -0.69 | -0.13 | -1 | FALSE | -0.43 | 0.12 | 0.09 | -0.03 | -0.04 |
| TCGA -BR- 7851-01A- 11R-2203-13 | FALSE | FALSE | 0.11 | 0.25 | 0.99 | 0.07 | 0.27 | -0.06 | FALSE | -0.1 | 0.11 | 0.01 | -0.01 | 0.02 |
| TCGA -CG-4306-01A-01R- 1157-13 | FALSE | FALSE | 0.09 | 0.41 | 0.99 | 0.17 | 0.85 | -0.16 | FALSE | -1.04 | 0.09 | 0.02 | 0.05 | -0.06 |
| TCGA -HU-A4GC -01A-12R -A251- 31 | FALSE | FALSE | 0.06 | -0.23 | 0.41 | -0.05 | -0.36 | 0.09 | FALSE | -0.83 | 0.06 | 0.03 | -0.07 | 0.05 |
| TCGA -VQ-A91D-01A- 11R-A414-31 | FALSE | FALSE | 0.04 | 1.07 | 1 | 0.46 | 1.15 | -0.87 | FALSE | -0.28 | 0.04 | 0.08 | 0.02 | -0.1 |
| TCGA -IN-AB1X-11A-21R-A39E- 31 | FALSE | FALSE | 0.03 | -1.97 | 0.97 | -1.51 | -0.07 | -1.18 | FALSE | -0.1 | 0.03 | -0.01 | 0 | 0.02 |
| TCGA -BR-4191-01A-02R-1131-13 | FALSE | FALSE | 0.02 | 1.09 | 0.19 | 0.73 | 0.36 | 0.51 | TRUE | 0.02 | -1.31 | -0.18 | 0 | -0.02 |
| TCGA -VQ-AA6A -01A-11R -A414- 31 | FALSE | FALSE | 0.02 | -1.04 | 0.09 | -0.86 | -0.73 | -1.15 | FALSE | -0.65 | 0.02 | 0.06 | -0.09 | 0.02 |
| TCGA -CG-4476-01A-01R- 1157-13 | FALSE | FALSE | 0.02 | -0.45 | 0.3 | -0.16 | -0.24 | 0.02 | FALSE | -0.04 | 0.02 | -0.14 | 0.1 | 0.04 |
| TCGA -F1-A72C-01A-21R -A33Y-31 | FALSE | FALSE | 0.01 | 0.3 | 0.26 | 0.32 | -0.08 | 0.18 | TRUE | 0.01 | 0.57 | 0.05 | 0.07 | -0.04 |
| TCGA -BR- 6452-01A- 12R-1802-13 | FALSE | FALSE | 0.01 | 1.01 | 0.99 | 0.62 | 0.42 | 0.1 | FALSE | -0.34 | 0.01 | 0 | 0.01 | 0 |

| TCGA -D7-6528-01A-11R -1802- 13 | FALSE | FALSE | 0.01 | -0.33 | 0.3 | -0.57 | 1.03 | -1.2 | FALSE | -0.19 | 0.01 | 0.14 | -0.15 | 0 |
| --- | --- | --- | --- | --- | --- | --- | --- | --- | --- | --- | --- | --- | --- | --- |
| TCGA -VQ-AA6K-01A- 11R-A414-31 | FALSE | FALSE | 0 | 0.14 | 0.11 | 0.08 | -0.22 | 0.4 | FALSE | -0.03 | 0 | 0.04 | 0.01 | -0.04 |
| TCGA -BR- 8589-01A- 11R-2402-13 | FALSE | TRUE | 0 | 2.91 | 0.41 | 1.77 | 0.9 | 2.79 | TRUE | 0 | -2.51 | -0.06 | -0.28 | -0.04 |
| TCGA -BR-A4QL-01A-31R -A251- 31 | FALSE | TRUE | -0.03 | -0.27 | 1 | -0.29 | 0.03 | 0.02 | FALSE | -1.04 | -0.03 | 0.1 | -0.2 | 0.08 |
| TCGA -IN-A7NR -01A-11R -A354-31 | FALSE | TRUE | -0.04 | -0.33 | 0.52 | -0.31 | -0.42 | 0.06 | FALSE | 0.41 | -0.04 | 0.02 | -0.05 | 0.01 |
| TCGA -BR- 8485-01A- 11R-2402-13 | FALSE | TRUE | -0.05 | 0.11 | 0.03 | 0.19 | -0.29 | 0.89 | TRUE | -0.05 | 0.86 | 0.06 | 0.06 | 0.02 |
| TCGA -RD-A8NB-01A-12R -A39E- 31 | FALSE | TRUE | -0.05 | 1.79 | 0.92 | 0.87 | 0.95 | 0.04 | FALSE | 0.35 | -0.05 | 0 | 0.07 | -0.08 |
| TCGA -D7-8573-01A-11R -2343- 13 | FALSE | TRUE | -0.06 | 0.61 | 0.33 | -0.24 | 0.15 | -0.47 | FALSE | -0.75 | -0.06 | 0.13 | -0.19 | 0.03 |
| TCGA -HU-A4H2-01A- 11R-A251-31 | FALSE | TRUE | -0.06 | 0.33 | 0.44 | 0.1 | -0.08 | 0.18 | FALSE | -0.43 | -0.06 | 0.07 | -0.12 | 0.02 |
| TCGA -VQ-A924-01A-11R -A414- 31 | FALSE | TRUE | -0.08 | 1.23 | 1 | 0.7 | 1.45 | 0.35 | TRUE | -0.08 | -0.63 | 0.06 | -0.08 | -0.07 |
| TCGA -ZA-A8F6 -01A-23R -A36D-31 | FALSE | TRUE | -0.09 | -1.05 | 0.38 | -0.42 | -0.29 | -0.65 | FALSE | 0.75 | -0.09 | -0.17 | 0.15 | 0.02 |
| TCGA -SW-A7EA-01A-12R -A354-31 | FALSE | TRUE | -0.09 | 1.82 | 0.99 | 1.07 | 2.79 | 0.42 | TRUE | -0.09 | 0.48 | 0.13 | 0.02 | -0.09 |
| TCGA -HU-A4H4-01A-21R-A251-31 | FALSE | TRUE | -0.1 | 2.34 | 0.81 | 1.36 | 1.12 | 0.98 | TRUE | -0.1 | -2.15 | 0 | -0.25 | -0.08 |
| TCGA -SW-A7EB-01A- 11R-A354-31 | FALSE | TRUE | -0.12 | -0.52 | 0.14 | -0.21 | -0.58 | 0.47 | FALSE | 0.23 | -0.12 | 0.02 | -0.08 | 0.03 |
| TCGA -CG-4477-01A-01R- 1157-13 | FALSE | TRUE | -0.12 | 2.36 | 0.64 | 1 | 1.79 | 0.32 | TRUE | -0.12 | -1.13 | -0.04 | -0.05 | -0.08 |
| TCGA -CG-4305-01A-01R- 1157-13 | FALSE | TRUE | -0.13 | 1.39 | 0.95 | 0.75 | 0.66 | 0.59 | TRUE | -0.13 | 0.1 | -0.08 | 0.06 | 0.03 |
| TCGA -CG-4301-01A-01R- 1157-13 | FALSE | TRUE | -0.13 | 0.08 | 0.32 | -0.42 | 0.28 | -1.07 | FALSE | -0.79 | -0.13 | -0.09 | 0.09 | -0.02 |
| TCGA -VQ-A8E3-01A- 11R-A39E-31 | FALSE | TRUE | -0.14 | 2.04 | 0.94 | 1.12 | 2.36 | 0.43 | TRUE | -0.14 | -1.64 | 0.04 | -0.25 | -0.05 |
| TCGA -CD-A48A-01A- 12R-A36D- 31 | FALSE | TRUE | -0.15 | -0.15 | 0.15 | -0.17 | 2.22 | -0.55 | FALSE | 0.03 | -0.15 | 0.04 | -0.08 | 0.01 |
| TCGA -BR-4253-01A-01R-1131-13 | FALSE | TRUE | -0.16 | 4.73 | 0.9 | 2.51 | 3.22 | 2.44 | TRUE | -0.16 | -2.96 | -0.13 | -0.2 | -0.12 |
| TCGA -IN-A6RL -01A-11R -A32D-31 | FALSE | TRUE | -0.16 | 0.06 | 0.34 | -0.26 | -0.49 | -0.74 | FALSE | -0.47 | -0.16 | 0.04 | -0.09 | 0 |
| TCGA -HU-8249-01A-11R -A36D-31 | FALSE | TRUE | -0.17 | 0.61 | 0.1 | 0.03 | 0.63 | 0.23 | TRUE | -0.17 | -0.48 | 0.1 | -0.18 | 0.01 |
| TCGA -FP-7735-01A-11R -2055-13 | FALSE | TRUE | -0.18 | -0.09 | 0.03 | 0.4 | -0.45 | 0 | FALSE | 0.36 | -0.18 | -0.03 | -0.05 | 0.05 |
| TCGA -BR-4357-01A-01R-1157-13 | FALSE | TRUE | -0.18 | 1.83 | 0.36 | 0.79 | 1.31 | 0.45 | TRUE | -0.18 | -0.48 | -0.02 | 0 | -0.06 |
| TCGA -KB-A6F7 -01A-12R -A32D-31 | FALSE | TRUE | -0.19 | 1.11 | 0.25 | 0.88 | -0.08 | 1.31 | TRUE | -0.19 | -0.5 | 0.06 | -0.15 | 0 |
| TCGA -IN-A6RN-01A-12R -A33Y- 31 | FALSE | TRUE | -0.2 | -1.22 | 0.61 | -0.94 | 0.26 | -1 | FALSE | -0.36 | -0.2 | 0.02 | -0.07 | 0.01 |
| TCGA -F1-A448-01A- 11R-A24K- 31 | FALSE | TRUE | -0.2 | -0.74 | 0.91 | -0.11 | 0.16 | -0.46 | FALSE | 0.56 | -0.2 | -0.07 | 0.09 | -0.05 |
| TCGA -CG- 5726-01A- 11R- 1602-13 | FALSE | TRUE | -0.21 | -0.1 | 1 | -0.44 | -0.06 | -0.7 | FALSE | -0.73 | -0.21 | 0.14 | -0.24 | 0.06 |
| TCGA -BR- 8682-01A- 11R-2402-13 | FALSE | TRUE | -0.22 | -1.09 | 0.34 | -0.53 | -0.45 | -0.39 | FALSE | 0.68 | -0.22 | -0.16 | 0.12 | 0.03 |
| TCGA -BR- 6455-01A- 11R-1802-13 | FALSE | TRUE | -0.22 | 1.24 | 0.49 | 0.76 | -0.13 | 1.55 | TRUE | -0.22 | 0.15 | 0.03 | -0.05 | 0.03 |
| TCGA -VQ-A8P8-01A-11R-A39E-31 | FALSE | TRUE | -0.22 | -0.89 | 0.63 | -0.28 | -0.34 | -0.41 | FALSE | 0.36 | -0.22 | -0.03 | 0 | 0 |
| TCGA -BR- 8077-01A- 11R-2343-13 | FALSE | TRUE | -0.24 | 1.13 | 0.08 | 0.57 | 0.29 | 1.76 | TRUE | -0.24 | -0.39 | 0.01 | -0.08 | 0 |
| TCGA -IN-7806-11A-01R -2055-13 | FALSE | TRUE | -0.24 | -1.89 | 0.99 | -1.05 | 0.01 | -0.08 | FALSE | 0.13 | -0.24 | -0.01 | -0.06 | 0.04 |
| TCGA -RD-A7BS-01A-11R -A32D-31 | FALSE | TRUE | -0.26 | 0.96 | 0.47 | 0.08 | 0.38 | -0.42 | FALSE | 0.77 | -0.26 | 0 | 0.04 | -0.07 |
| TCGA -BR- 8591-01A- 11R-2402-13 | FALSE | TRUE | -0.26 | 1.27 | 0.99 | 0.39 | 0.43 | -0.74 | FALSE | -0.34 | -0.26 | 0.04 | -0.02 | -0.06 |
| TCGA -BR-4366-01A-01R-1157-13 | FALSE | TRUE | -0.27 | -0.36 | 0.5 | -0.9 | -0.49 | 0.15 | FALSE | -1.48 | -0.27 | -0.02 | -0.04 | 0.03 |
| TCGA -BR-A4J4-01A- 12R-A251-31 | FALSE | TRUE | -0.29 | 1.01 | 0.45 | 0.28 | 1.22 | 0.04 | FALSE | -0.33 | -0.29 | -0.02 | 0 | -0.03 |

| TCGA -IN-7806-01A-11R -2055-13 | FALSE | TRUE | -0.29 | -1.73 | 0.43 | -1.16 | -0.82 | -1.21 | FALSE | -0.26 | -0.29 | 0.02 | -0.13 | 0.06 |
| --- | --- | --- | --- | --- | --- | --- | --- | --- | --- | --- | --- | --- | --- | --- |
| TCGA -BR- 8361-01A- 11R-2343-13 | FALSE | TRUE | -0.3 | 1.38 | 1 | 1.04 | 1.33 | 1.57 | TRUE | -0.3 | -0.89 | 0.06 | -0.19 | -0.01 |
| TCGA -RD-A7BT-01A-11R -A33Y-31 | FALSE | TRUE | -0.3 | -0.42 | 0.37 | -0.47 | -0.57 | -0.47 | FALSE | -1.28 | -0.3 | 0.12 | -0.26 | 0.07 |
| TCGA -ZQ-A9CR-01A-11R-A39E-31 | FALSE | TRUE | -0.3 | 1.06 | 0.09 | 0.54 | 0.76 | 0.38 | TRUE | -0.3 | 1.83 | 0.12 | 0.23 | -0.08 |
| TCGA -VQ-A8PP-01A-21R-A414-31 | FALSE | TRUE | -0.3 | 0.73 | 0.98 | 0.36 | 0.33 | 0.37 | TRUE | -0.3 | 0.74 | 0.06 | 0.09 | -0.04 |
| TCGA -HU-A4GX-01A- 12R-A251-31 | FALSE | TRUE | -0.3 | 2.05 | 0.98 | 1.34 | 0.62 | 1.93 | TRUE | -0.3 | -1.97 | -0.01 | -0.25 | -0.06 |
| TCGA -VQ-A8PU-01A-12R-A414- 31 | FALSE | TRUE | -0.32 | -1.67 | 0.3 | -1.12 | -0.63 | -1.22 | FALSE | -0.98 | -0.32 | 0.08 | -0.16 | 0.03 |
| TCGA -D7-A6EZ-01A-11R -A31P- 31 | FALSE | TRUE | -0.33 | 3.29 | 0.61 | 1.7 | 1.65 | 1.07 | TRUE | -0.33 | -0.76 | 0.01 | -0.05 | -0.08 |
| TCGA -FP-8211-01A-11R -2343-13 | FALSE | TRUE | -0.34 | 0.17 | 0.45 | 0.18 | -0.02 | 0.46 | FALSE | -0.22 | -0.34 | 0.04 | -0.12 | 0.02 |
| TCGA -VQ-A91E-01A- 31R-A414-31 | FALSE | TRUE | -0.34 | 0.17 | 0.99 | 0.24 | 0.15 | 0.44 | TRUE | -0.34 | -1.78 | 0.03 | -0.27 | -0.03 |
| TCGA -HU-A4H0-01A- 11R-A251-31 | FALSE | TRUE | -0.34 | 3.29 | 0.29 | 1.7 | 2.64 | 1.02 | TRUE | -0.34 | -1.89 | 0.02 | -0.26 | -0.05 |
| TCGA -BR-4294-01A-01R-1131-13 | FALSE | TRUE | -0.35 | -0.97 | 0.65 | -0.9 | 0.35 | -0.48 | FALSE | -0.71 | -0.35 | -0.05 | -0.06 | 0.06 |
| TCGA -BR- 7722-01A- 31R-2203-13 | FALSE | TRUE | -0.36 | -0.72 | 0.25 | -0.3 | -0.49 | -0.46 | FALSE | 0.94 | -0.36 | -0.07 | 0.03 | -0.01 |
| TCGA -BR-4267-01A-01R-1131-13 | FALSE | TRUE | -0.36 | -0.26 | 0.71 | -0.24 | 0.79 | -0.55 | FALSE | -0.85 | -0.36 | -0.01 | -0.04 | -0.01 |
| TCGA -IN-8663-11A-01R -2402-13 | FALSE | TRUE | -0.37 | -1.4 | 0.99 | -1.36 | 0.46 | -1.04 | FALSE | -0.45 | -0.37 | -0.01 | -0.08 | 0.04 |
| TCGA -HU-A4GU-01A- 11R-A251-31 | FALSE | TRUE | -0.39 | -1.1 | 0.9 | -0.94 | -0.31 | -0.88 | FALSE | -1.03 | -0.39 | 0.09 | -0.15 | 0 |
| TCGA -IN-A6RI -01A-11R -A32D-31 | FALSE | TRUE | -0.39 | -1.05 | 0.75 | -0.9 | -0.43 | -1.18 | FALSE | -0.92 | -0.39 | 0.09 | -0.22 | 0.05 |
| TCGA -VQ-A91N-01A- 11R-A414-31 | FALSE | TRUE | -0.39 | -0.06 | 0.66 | -0.28 | -0.3 | -0.47 | FALSE | -0.82 | -0.39 | 0.1 | -0.19 | 0.02 |
| TCGA -VQ-A8P3-01A-11R-A36D-31 | FALSE | TRUE | -0.4 | -0.28 | 0.86 | 0.28 | 0.47 | 0.54 | TRUE | -0.4 | 1.17 | 0.12 | 0.02 | 0.03 |
| TCGA -KB-A93H-01A- 11R-A39E-31 | FALSE | TRUE | -0.42 | 0.31 | 0.48 | -0.56 | -0.14 | 1.06 | FALSE | -1.36 | -0.42 | 0.14 | -0.26 | 0.03 |
| TCGA -BR- 6802-11A-01R-1884-13 | FALSE | TRUE | -0.42 | -0.36 | 1 | 0.3 | 0.02 | 1.96 | TRUE | -0.42 | -0.52 | -0.06 | -0.09 | 0.07 |
| TCGA -IN-A6RR-01A- 12R-A32D- 31 | FALSE | TRUE | -0.44 | -0.84 | 0.41 | -0.38 | -0.49 | -0.2 | FALSE | -0.61 | -0.44 | 0.04 | -0.14 | 0.02 |
| TCGA -D7-A4YX-01A- 11R-A251-31 | FALSE | TRUE | -0.44 | 3.03 | 0.74 | 1.53 | 1.9 | 1.19 | TRUE | -0.44 | -1.42 | 0.03 | -0.19 | -0.06 |
| TCGA -BR-4279-01A-01R-1131-13 | FALSE | TRUE | -0.46 | 0.57 | 0.88 | 0.34 | 0.43 | 0.97 | TRUE | -0.46 | 0.84 | -0.1 | 0.24 | -0.01 |
| TCGA -BR-4367-01A-01R-1157-13 | FALSE | TRUE | -0.46 | 2.09 | 0.44 | 0.93 | 0.63 | 0.21 | TRUE | -0.46 | 0.12 | -0.05 | 0.14 | -0.07 |
| TCGA -VQ-A925-01A-11R -A414- 31 | FALSE | TRUE | -0.47 | -0.73 | 0.35 | -0.6 | -0.27 | -0.3 | FALSE | -0.27 | -0.47 | 0.04 | -0.14 | 0.03 |
| TCGA -BR-4368-01A-01R-1157-13 | FALSE | TRUE | -0.48 | 1.37 | 0.99 | 0.8 | 0.65 | 0.99 | TRUE | -0.48 | 0.33 | 0.01 | 0.06 | -0.03 |
| TCGA -BR-A4PF-01A-11R -A251- 31 | FALSE | TRUE | -0.48 | 1.18 | 0.53 | 0.57 | 1.28 | 0.71 | TRUE | -0.48 | -1.86 | -0.04 | -0.16 | -0.08 |
| TCGA -HU-A4G3-01A- 11R-A24K- 31 | FALSE | TRUE | -0.53 | -1.37 | 0.83 | -0.87 | -0.54 | -0.85 | FALSE | -0.16 | -0.53 | -0.02 | -0.11 | 0.04 |
| TCGA -D7-8576-01A-11R -2343- 13 | FALSE | TRUE | -0.53 | -0.25 | 0.26 | -0.57 | -0.32 | -0.97 | FALSE | -0.17 | -0.53 | -0.03 | -0.07 | 0.03 |
| TCGA -BR-A44T-01A- 32R-A24K- 31 | FALSE | TRUE | -0.54 | 0.64 | 0.38 | 0.67 | 0.32 | 0.98 | FALSE | 1.34 | -0.54 | -0.13 | 0.02 | 0.02 |
| TCGA -IN-A6RJ -01A-21R -A33Y-31 | FALSE | TRUE | -0.56 | -0.7 | 0.94 | -0.65 | 0.29 | -0.58 | FALSE | 0.2 | -0.56 | 0 | -0.1 | 0.01 |
| TCGA -VQ-A91U-01A-11R -A414- 31 | FALSE | TRUE | -0.57 | 1.5 | 0.39 | 0.65 | 0.07 | 0.77 | TRUE | -0.57 | -1.51 | 0 | -0.23 | 0.01 |
| TCGA -BR- 8486-01A- 31R-2402-13 | FALSE | TRUE | -0.58 | 0.64 | 0.11 | 0.09 | 0.14 | -0.98 | FALSE | 0.61 | -0.58 | -0.02 | -0.03 | -0.03 |
| TCGA -CD- 8534-01A- 11R-2343-13 | FALSE | TRUE | -0.59 | -0.25 | 0.18 | -0.19 | -0.38 | -0.45 | FALSE | 0.12 | -0.59 | -0.02 | -0.11 | 0.04 |
| TCGA -CG- 5718-01A- 11R- 1602-13 | FALSE | TRUE | -0.61 | 0.36 | 0.14 | 0.32 | 0.1 | -0.21 | FALSE | 0.96 | -0.61 | -0.02 | -0.07 | -0.01 |
| TCGA -HU-A4GT-01A-21R-A251-31 | FALSE | TRUE | -0.61 | -0.24 | 1 | -0.37 | 0.28 | -0.46 | FALSE | -1.37 | -0.61 | 0.09 | -0.22 | 0.03 |

| TCGA -VQ-A91S-01A- 11R-A414-31 | FALSE | TRUE | -0.61 | 1.46 | 0.28 | 0.67 | 1.53 | 1.57 | TRUE | -0.61 | -0.91 | 0.06 | -0.15 | -0.05 |
| --- | --- | --- | --- | --- | --- | --- | --- | --- | --- | --- | --- | --- | --- | --- |
| TCGA -IN-A6RS -01A-12R -A354-31 | FALSE | TRUE | -0.61 | 0.44 | 0.61 | 0.35 | 0.12 | 0.5 | TRUE | -0.61 | -0.92 | 0.03 | -0.17 | -0.01 |
| TCGA -VQ-A8PM -01A-21R -A414- 31 | FALSE | TRUE | -0.62 | 0.12 | 0.16 | 0.26 | -0.19 | -0.06 | FALSE | 0.69 | -0.62 | -0.08 | -0.02 | 0 |
| TCGA -HU-A4GF-01A-11R -A24K-31 | FALSE | TRUE | -0.65 | 0.37 | 0.08 | -0.23 | -0.04 | -0.04 | FALSE | -0.59 | -0.65 | 0.08 | -0.21 | 0.02 |
| TCGA -R5-A7ZR-01A- 11R-A354-31 | FALSE | TRUE | -0.66 | 0.02 | 0.47 | -0.34 | -0.24 | -0.04 | FALSE | -0.72 | -0.66 | 0.06 | -0.21 | 0.03 |
| TCGA -VQ-AA69-01A-11R -A414- 31 | FALSE | TRUE | -0.67 | 0.86 | 0.51 | 0.51 | 0.95 | 0.15 | FALSE | -0.85 | -0.67 | 0.07 | -0.16 | -0.01 |
| TCGA -CG-4444-01A-01R- 1157-13 | FALSE | TRUE | -0.68 | -0.24 | 0.39 | -0.15 | 0.01 | -0.63 | FALSE | -0.69 | -0.68 | -0.03 | -0.06 | -0.01 |
| TCGA -BR- 8487-01A- 11R-2402-13 | FALSE | TRUE | -0.71 | 0.97 | 0.99 | 0.28 | 0.38 | -0.39 | FALSE | -0.89 | -0.71 | 0.01 | -0.14 | 0 |
| TCGA -D7-8574-01A-13R -2343- 13 | FALSE | TRUE | -0.72 | -0.09 | 0.09 | 0.6 | -0.3 | 1.43 | FALSE | 1.89 | -0.72 | -0.2 | 0.09 | 0.02 |
| TCGA -BR- 8368-01A- 11R-2343-13 | FALSE | TRUE | -0.72 | 0.11 | 1 | 0.1 | -0.13 | 0.08 | FALSE | -0.87 | -0.72 | 0.1 | -0.25 | 0.03 |
| TCGA -VQ-A8DT-01A- 11R-A36D- 31 | FALSE | TRUE | -0.73 | -0.96 | 0.92 | -0.57 | -0.3 | -0.26 | FALSE | -1.13 | -0.73 | 0.05 | -0.21 | 0.05 |
| TCGA -D7-A74A -01A-11R -A32D-31 | FALSE | TRUE | -0.73 | 0.46 | 0.67 | 0.08 | -0.24 | -0.08 | FALSE | -0.87 | -0.73 | 0.08 | -0.24 | 0.05 |
| TCGA -BR-4363-01A-01R-1157-13 | FALSE | TRUE | -0.75 | 1.87 | 0.89 | 0.81 | 0.57 | 0.41 | TRUE | -0.75 | 0.35 | -0.02 | 0.12 | -0.06 |
| TCGA -VQ-A91X -01A-12R -A414- 31 | FALSE | TRUE | -0.75 | -1.7 | 0.55 | -1.2 | -0.49 | -1.02 | FALSE | -1.03 | -0.75 | 0.09 | -0.28 | 0.07 |
| TCGA -IN-AB1V-01A-21R-A414-31 | FALSE | TRUE | -0.78 | -1.39 | 0.8 | -0.96 | -0.05 | -0.64 | FALSE | -0.02 | -0.78 | -0.02 | -0.1 | 0 |
| TCGA -HU-A4GP-01A-11R -A251-31 | FALSE | TRUE | -0.79 | -1.22 | 0.78 | -0.81 | -0.19 | -0.5 | FALSE | -0.54 | -0.79 | 0.04 | -0.17 | 0 |
| TCGA -F1- 6177-01A- 11R-1802-13 | FALSE | TRUE | -0.81 | -0.08 | 1 | -0.31 | 0.08 | -0.77 | FALSE | -0.43 | -0.81 | 0.07 | -0.19 | -0.01 |
| TCGA -VQ-AA68-01A-11R -A414- 31 | FALSE | TRUE | -0.86 | 0.59 | 0.39 | 0.24 | 0.22 | 0.34 | FALSE | -0.19 | -0.86 | 0.04 | -0.16 | -0.02 |
| TCGA -CG-4465-01A-01R- 1157-13 | FALSE | TRUE | -0.87 | 0.87 | 0.99 | 0.22 | 0.66 | -0.66 | FALSE | -0.89 | -0.87 | -0.06 | -0.04 | -0.03 |
| TCGA -BR-4280-01A-01R-1131-13 | FALSE | TRUE | -0.88 | 1.15 | 0.99 | 0.56 | 0.86 | 0.2 | TRUE | -0.88 | -1.62 | 0.01 | -0.26 | 0 |
| TCGA -HF-7134-01A-11R-2055- 13 | FALSE | TRUE | -0.91 | -0.62 | 0.49 | -0.43 | 0.05 | -0.67 | FALSE | -0.32 | -0.91 | 0.03 | -0.14 | -0.02 |
| TCGA -IN-A6RO-01A- 12R-A33Y-31 | FALSE | TRUE | -0.94 | 0.54 | 0.71 | -0.16 | -0.44 | -0.82 | FALSE | -1.01 | -0.94 | 0.09 | -0.27 | 0.03 |
| TCGA -VQ-A927-01A-12R -A414- 31 | FALSE | TRUE | -0.95 | -0.8 | 0.69 | -0.52 | -0.23 | -0.76 | FALSE | 0.27 | -0.95 | -0.08 | -0.06 | 0.01 |
| TCGA -BR-4257-01A-01R-1131-13 | FALSE | TRUE | -0.95 | 0.98 | 0.99 | 0.58 | 0.5 | 0.42 | TRUE | -0.95 | 0.27 | -0.02 | 0.08 | -0.01 |
| TCGA -CG- 5720-01A- 11R- 1602-13 | FALSE | TRUE | -0.95 | 0.26 | 0.9 | 0.34 | 0.32 | 0.08 | FALSE | 1.02 | -0.95 | -0.06 | -0.06 | -0.01 |
| TCGA -CG-4304-01A-01R- 1157-13 | FALSE | TRUE | -0.96 | 0.33 | 0.35 | 0.1 | 0.54 | -0.27 | FALSE | 0.1 | -0.96 | -0.14 | 0.03 | -0.02 |
| TCGA -D7-A4Z0-01A-22R-A251-31 | FALSE | TRUE | -0.96 | -0.8 | 0.49 | -0.31 | 0.19 | -0.51 | FALSE | 1.08 | -0.96 | -0.16 | 0.06 | -0.03 |
| TCGA -HU-A4GD-01A-11R-A36D-31 | FALSE | TRUE | -0.97 | -1.8 | 0.1 | -1.23 | -0.59 | -0.27 | FALSE | -0.67 | -0.97 | 0.05 | -0.26 | 0.05 |
| TCGA -HU-A4GH-01A-11R-A24K-31 | FALSE | TRUE | -1 | -0.95 | 0.39 | -0.78 | -0.17 | -0.74 | FALSE | -0.64 | -1 | 0.06 | -0.22 | 0 |
| TCGA -HU-A4H6-01A- 11R-A251-31 | FALSE | TRUE | -1.02 | -0.09 | 0.19 | -0.21 | -0.09 | -0.35 | FALSE | 0.17 | -1.02 | 0.03 | -0.18 | -0.02 |
| TCGA -CG-4438-01A-01R- 1157-13 | FALSE | TRUE | -1.04 | 2.19 | 0.12 | 0.85 | 0.92 | 0.15 | FALSE | -0.6 | -1.04 | 0 | -0.13 | -0.03 |
| TCGA -BR-4361-01A-01R-1157-13 | FALSE | TRUE | -1.09 | 1.39 | 0.99 | 0.72 | 1.23 | 0.33 | TRUE | -1.09 | 0.5 | 0.01 | 0.11 | -0.05 |
| TCGA -HF-A5NB-01A- 11R-A31P -31 | FALSE | TRUE | -1.1 | 0.11 | 1 | -0.02 | 0.46 | 0.07 | FALSE | -0.63 | -1.1 | 0.05 | -0.23 | 0.01 |
| TCGA -BR- 8372-01A- 11R-2343-13 | FALSE | TRUE | -1.11 | 2.75 | 1 | 1.62 | 1.3 | 1.21 | TRUE | -1.11 | -1.26 | 0.01 | -0.19 | -0.02 |
| TCGA -HU-A4G2-01A- 11R-A251-31 | FALSE | TRUE | -1.13 | 1.46 | 0.44 | 0.36 | 0.14 | 0.47 | TRUE | -1.13 | -0.16 | 0.06 | -0.14 | 0.05 |
| TCGA -CD- 8531-01A- 11R-2343-13 | FALSE | TRUE | -1.14 | 0.05 | 0.48 | 0.6 | -0.1 | -0.6 | FALSE | 0.68 | -1.14 | -0.09 | -0.12 | 0.04 |
| TCGA -BR-4371-01A-01R-1157-13 | FALSE | TRUE | -1.16 | 0.49 | 0.82 | -0.15 | 0.44 | -0.63 | FALSE | -1.21 | -1.16 | 0.02 | -0.21 | 0.01 |

| TCGA -CG- 5730- 11A-01R- 1602-13 | FALSE | TRUE | -1.17 | -0.59 | 0.99 | 0.12 | 0.26 | 0.24 | FALSE | 1.03 | -1.17 | -0.11 | -0.08 | 0.02 |
| --- | --- | --- | --- | --- | --- | --- | --- | --- | --- | --- | --- | --- | --- | --- |
| TCGA -VQ-A8E0-01A- 11R-A414-31 | FALSE | TRUE | -1.18 | -0.54 | 0.33 | -0.26 | -0.26 | -1.2 | FALSE | -0.28 | -1.18 | -0.01 | -0.18 | 0.01 |
| TCGA -VQ-A91V -01A-11R -A414- 31 | FALSE | TRUE | -1.18 | -1.28 | 0.57 | -0.96 | -0.39 | -0.85 | FALSE | -0.64 | -1.18 | 0.03 | -0.2 | -0.02 |
| TCGA -HU-A4G8-01A- 11R-A251-31 | FALSE | TRUE | -1.2 | 1.96 | 1 | 0.86 | 1.43 | 0 | FALSE | -0.66 | -1.2 | 0.08 | -0.2 | -0.07 |
| TCGA -VQ-A91K-01A- 11R-A414-31 | FALSE | TRUE | -1.21 | 0 | 0.97 | 0.02 | 0.18 | -0.2 | FALSE | 0.82 | -1.21 | -0.08 | -0.06 | -0.04 |
| TCGA -HF-7131-01A-11R-2055- 13 | FALSE | TRUE | -1.23 | -0.04 | 0.4 | 0.24 | 0.61 | 0.14 | FALSE | 0.27 | -1.23 | -0.02 | -0.1 | -0.05 |
| TCGA -RD-A8N0-01A-12R -A36D-31 | FALSE | TRUE | -1.27 | 0.41 | 0.52 | 0.7 | 0.27 | 1.47 | FALSE | 1.09 | -1.27 | -0.18 | -0.02 | 0.01 |
| TCGA -CG-4436-01A-01R- 1157-13 | FALSE | TRUE | -1.28 | 0.87 | 0.52 | -0.25 | 1.27 | -0.42 | FALSE | -1.54 | -1.28 | 0.03 | -0.22 | 0 |
| TCGA -HU-8610-01A-22R -2402- 13 | FALSE | TRUE | -1.29 | -0.06 | 0.08 | 0.12 | 0.84 | -0.83 | FALSE | 0.78 | -1.29 | -0.11 | -0.02 | -0.04 |
| TCGA -VQ-AA6F-01A-31R-A414- 31 | FALSE | TRUE | -1.29 | 0.56 | 0.14 | 0.2 | -0.08 | -0.07 | FALSE | 0.23 | -1.29 | 0 | -0.17 | -0.04 |
| TCGA -BR- 6457-11A-01R-1802-13 | FALSE | TRUE | -1.29 | -0.85 | 0.98 | 0.45 | -0.25 | 1.4 | FALSE | 1.35 | -1.29 | -0.15 | -0.06 | 0.02 |
| TCGA -VQ-AA6G-01A- 11R-A414-31 | FALSE | TRUE | -1.34 | -0.91 | 0.64 | -0.55 | 0.83 | -1.04 | FALSE | -0.21 | -1.34 | 0 | -0.13 | -0.06 |
| TCGA -HU-8602-01A-11R -2402- 13 | FALSE | TRUE | -1.34 | 2.08 | 1 | 1.34 | 1.99 | 0.12 | FALSE | -0.45 | -1.34 | 0.03 | -0.11 | -0.11 |
| TCGA -D7-A6F2-01A-12R-A31P-31 | FALSE | TRUE | -1.34 | -0.05 | 0.09 | 0.18 | 0 | 0.39 | FALSE | 0.31 | -1.34 | -0.1 | -0.12 | 0.01 |
| TCGA -HU-A4GY-01A-21R -A24K-31 | FALSE | TRUE | -1.35 | 1.29 | 0.12 | 1 | 0.33 | 1.02 | FALSE | 1.43 | -1.35 | -0.23 | 0.05 | -0.01 |
| TCGA -HU-A4G9-01A- 11R-A24K- 31 | FALSE | TRUE | -1.38 | -0.8 | 1 | -0.95 | -0.37 | -1.05 | FALSE | -1.28 | -1.38 | 0.04 | -0.3 | 0.04 |
| TCGA -BR- 8676-01A- 11R-2402-13 | FALSE | TRUE | -1.38 | 1.17 | 0.87 | 0.95 | 0.12 | 1.54 | TRUE | -1.38 | -0.61 | 0.04 | -0.21 | 0.05 |
| TCGA -VQ-A8PC-01A- 11R-A39E- 31 | FALSE | TRUE | -1.39 | -0.26 | 0.07 | -0.09 | -0.14 | -0.47 | FALSE | 0.63 | -1.39 | -0.11 | -0.06 | -0.04 |
| TCGA -VQ-AA6J -01A-11R -A414-31 | FALSE | TRUE | -1.42 | 1.5 | 0.13 | 0.81 | 0.8 | 0.31 | FALSE | 0.22 | -1.42 | -0.05 | -0.13 | -0.05 |
| TCGA -FP-A8CX-01A-11R-A36D-31 | FALSE | TRUE | -1.42 | -0.28 | 0.71 | -0.1 | 0.16 | -0.23 | FALSE | 0.33 | -1.42 | -0.04 | -0.19 | 0.01 |
| TCGA -CG- 5716-01A-21R- 1802-13 | FALSE | TRUE | -1.46 | -1.07 | 0.86 | 0.02 | -0.26 | 0.07 | FALSE | 1.03 | -1.46 | -0.07 | -0.21 | 0.04 |
| TCGA -HU-A4H8-01A- 11R-A251-31 | FALSE | TRUE | -1.47 | 0.19 | 1 | -0.11 | 0.26 | -0.58 | FALSE | -1.03 | -1.47 | 0.03 | -0.28 | 0.03 |
| TCGA -HU-8244-01A-11R -2343- 13 | FALSE | TRUE | -1.47 | -1.4 | 0.85 | -1.02 | -0.37 | -0.88 | FALSE | -0.76 | -1.47 | 0.03 | -0.33 | 0.07 |
| TCGA -HU-8238-01A-11R -2343- 13 | FALSE | TRUE | -1.59 | -0.09 | 0.49 | -0.25 | 0.26 | -0.24 | FALSE | 0.84 | -1.59 | -0.04 | -0.15 | -0.05 |
| TCGA -F1- 6874-01A- 11R-1884-13 | FALSE | TRUE | -1.62 | 0.75 | 0.88 | 0.33 | 0.36 | -0.03 | FALSE | 0.78 | -1.62 | -0.08 | -0.04 | -0.12 |
| TCGA -CG-4469-01A-01R- 1157-13 | FALSE | TRUE | -1.63 | -0.09 | 0.8 | -0.39 | 0.44 | -1.17 | FALSE | -1.12 | -1.63 | -0.04 | -0.21 | 0 |
| TCGA -R5-A805-01A-11R -A36D-31 | FALSE | TRUE | -1.74 | -0.14 | 0.38 | 0 | 0.64 | -0.08 | FALSE | 0.68 | -1.74 | -0.14 | -0.01 | -0.1 |
| TCGA -BR- 8296-01A- 11R-2343-13 | FALSE | TRUE | -1.77 | 0.04 | 0.15 | 0.15 | -0.26 | 0.36 | FALSE | 1.24 | -1.77 | -0.1 | -0.14 | -0.03 |
| TCGA -D7-8575-01A-11R -2343- 13 | FALSE | TRUE | -1.97 | -0.51 | 0.2 | -0.29 | 0.23 | -0.45 | FALSE | 0.63 | -1.97 | -0.18 | -0.05 | -0.05 |
| TCGA -VQ-A8PQ-01A-11R -A414-31 | FALSE | TRUE | -1.98 | 0.63 | 0.07 | 0.68 | 0.16 | 1.36 | FALSE | 1.97 | -1.98 | -0.21 | -0.05 | -0.02 |
| TCGA -IN-7808-01A-11R -2203-13 | FALSE | TRUE | -2.08 | 0.77 | 0.1 | 0.95 | 0.07 | 1.64 | FALSE | 1.42 | -2.08 | -0.13 | -0.19 | 0 |
| TCGA -VQ-A8PX-01A-12R-A414- 31 | FALSE | TRUE | -2.11 | 0.25 | 0.9 | 0.04 | -0.45 | 0 | FALSE | -0.35 | -2.11 | -0.01 | -0.28 | -0.02 |
| TCGA -KB-A93J -01A- 11R-A39E- 31 | FALSE | TRUE | -2.14 | 1.87 | 0.4 | 1.01 | 0.81 | -0.05 | FALSE | 0.56 | -2.14 | -0.13 | -0.17 | -0.03 |
| TCGA -CG- 5728- 11A-01R- 1602-13 | FALSE | TRUE | -2.32 | 0.65 | 0.87 | 1.27 | 0.32 | 1.45 | FALSE | 2.12 | -2.32 | -0.19 | -0.17 | 0 |
| TCGA -HU-A4GJ-01A- 11R-A251-31 | FALSE | TRUE | -2.52 | 0.16 | 0.35 | 0.92 | -0.01 | 1.8 | FALSE | 1.65 | -2.52 | -0.17 | -0.23 | 0.02 |
| TCGA -FP-A9TM-01A- 11R-A39E-31 | FALSE | TRUE | -2.71 | -0.36 | 0.32 | -0.22 | -0.27 | -0.54 | FALSE | 0.34 | -2.71 | -0.07 | -0.35 | 0 |
