# Supplementary figures and images for: Identification and Validation of a m5C RNA Modification-Related Gene Signature for Predicting Prognosis and Immunotherapeutic Efficiency of Gastric Cancer
Source: J Oncol. 2023 Mar 8;2023:9931419. doi: 10.1155/2023/9931419 (PMC10017215; doi:10.1155/2023/9931419)

# NMF rank survey

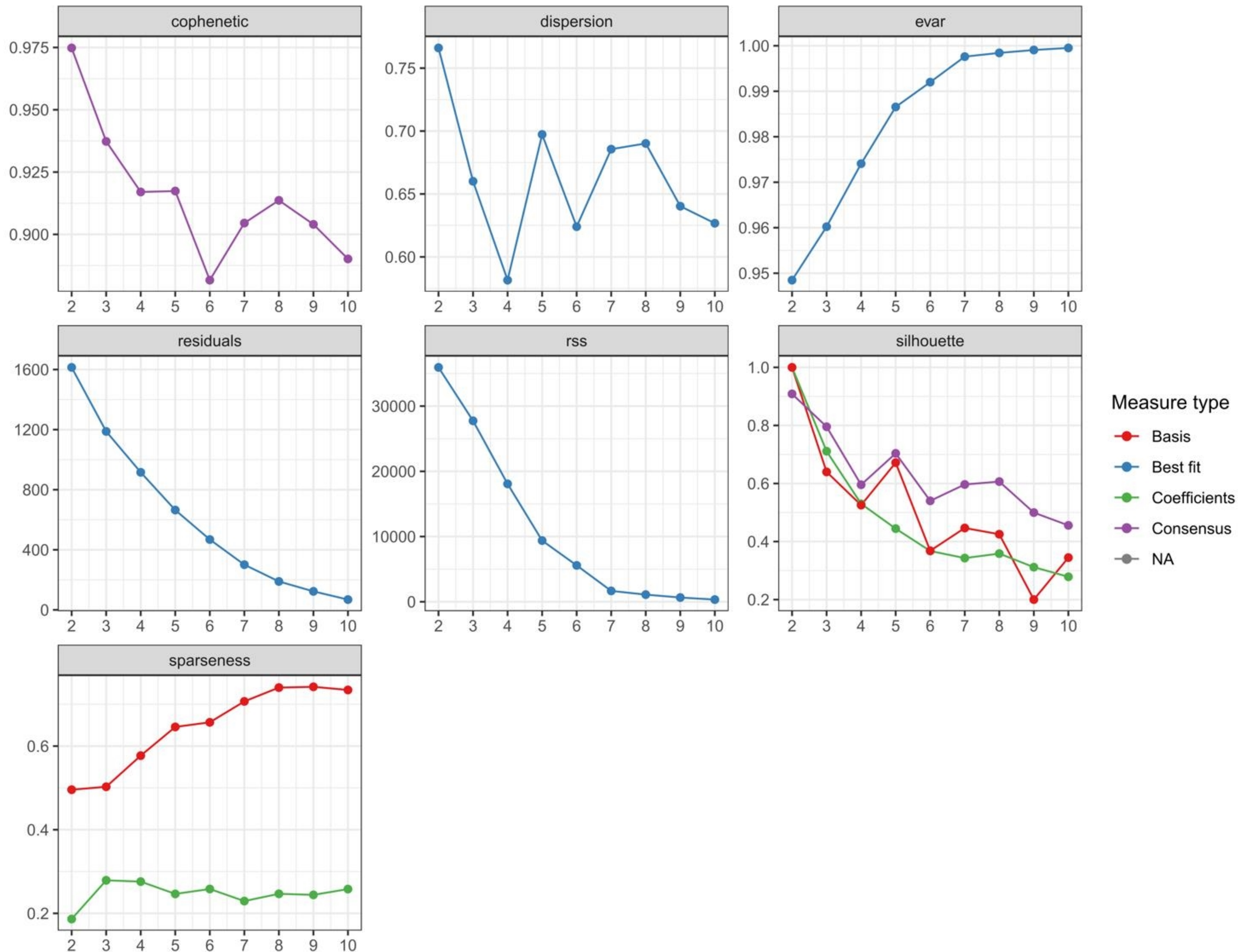

Factorization rank

Supplement: Supplementary Materials — Supplementary Figure 1: determination of the k value using the NMF rank survey with multiple parameters. Supplementary Figure 2: OS and DSS analyses of different subtypes. (a) Overall survival (OS) curves for 5 different subtypes. (b) Disease-specific survival (DSS) curves for 5 different subtypes. P < 0.05 showed statistically significant. Supplementary Figure 3: validation of the m5C-related risk model. (a) The KM survival curve of the high- and low-risk group in the validation set. (b) The KM survival curve of high- and low-risk group in the testing set. (c) The ROC curve and AUC for four signatures in the validation set. (d) The ROC curve and AUC of four signature in the test set. Supplementary Figure 4: the risk score, survival time, survival status, and expression of the four signatures in the training set (a), validation set (b), and testing set (c). Supplementary Figure 5: KM survival stratification analyses between high- and low-risk GC samples with clinicopathological data. Supplementary Table 1: the primer sequences for qRT-PCR. Supplementary Table 2: the detailed information for TIDE analysis. [file 9931419.f1.zip › Supplementary Figure 1.pdf]

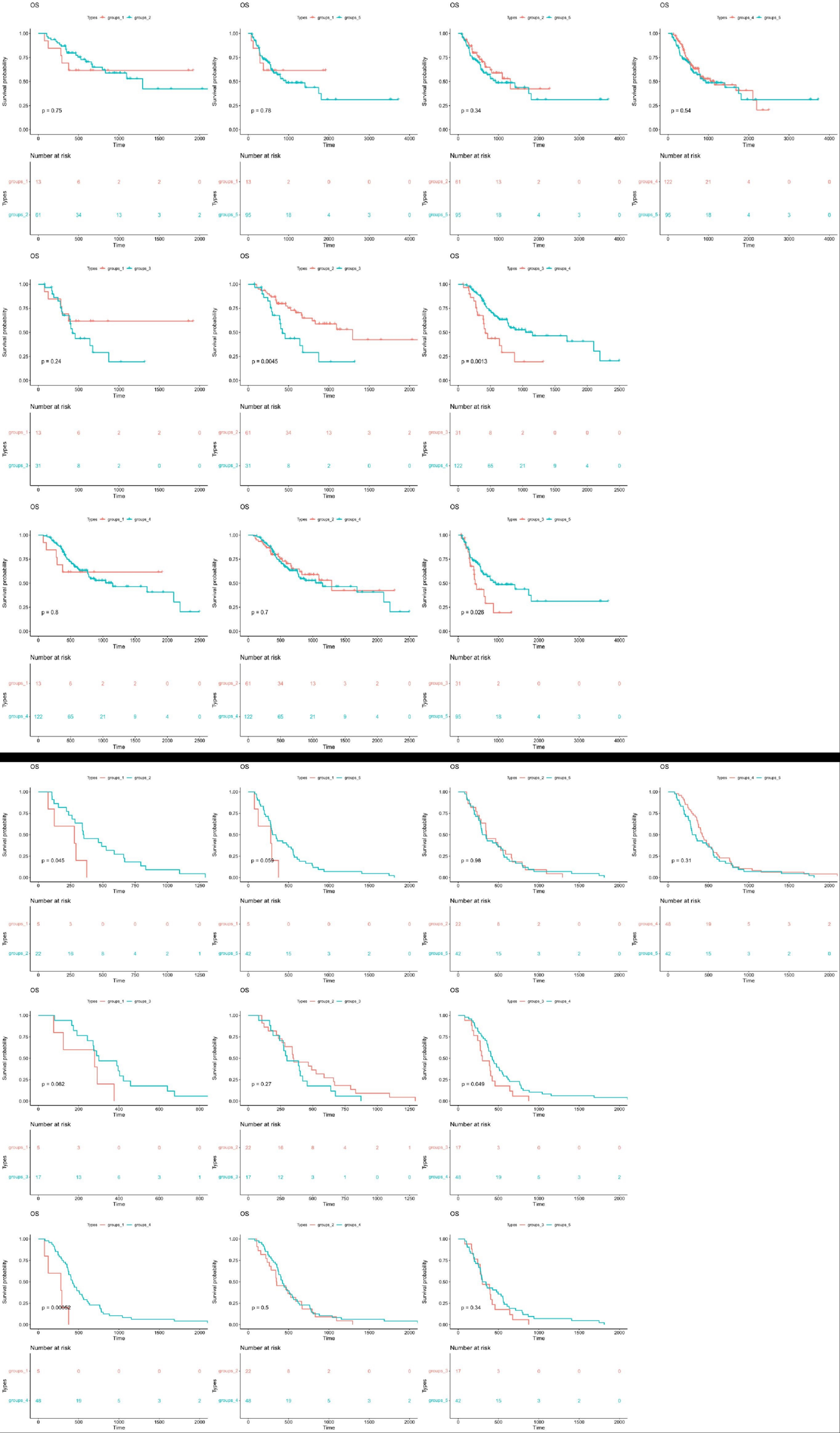

Supplement: Supplementary Materials — Supplementary Figure 1: determination of the k value using the NMF rank survey with multiple parameters. Supplementary Figure 2: OS and DSS analyses of different subtypes. (a) Overall survival (OS) curves for 5 different subtypes. (b) Disease-specific survival (DSS) curves for 5 different subtypes. P < 0.05 showed statistically significant. Supplementary Figure 3: validation of the m5C-related risk model. (a) The KM survival curve of the high- and low-risk group in the validation set. (b) The KM survival curve of high- and low-risk group in the testing set. (c) The ROC curve and AUC for four signatures in the validation set. (d) The ROC curve and AUC of four signature in the test set. Supplementary Figure 4: the risk score, survival time, survival status, and expression of the four signatures in the training set (a), validation set (b), and testing set (c). Supplementary Figure 5: KM survival stratification analyses between high- and low-risk GC samples with clinicopathological data. Supplementary Table 1: the primer sequences for qRT-PCR. Supplementary Table 2: the detailed information for TIDE analysis. [file 9931419.f1.zip › Supplementary Figure 2.pdf]

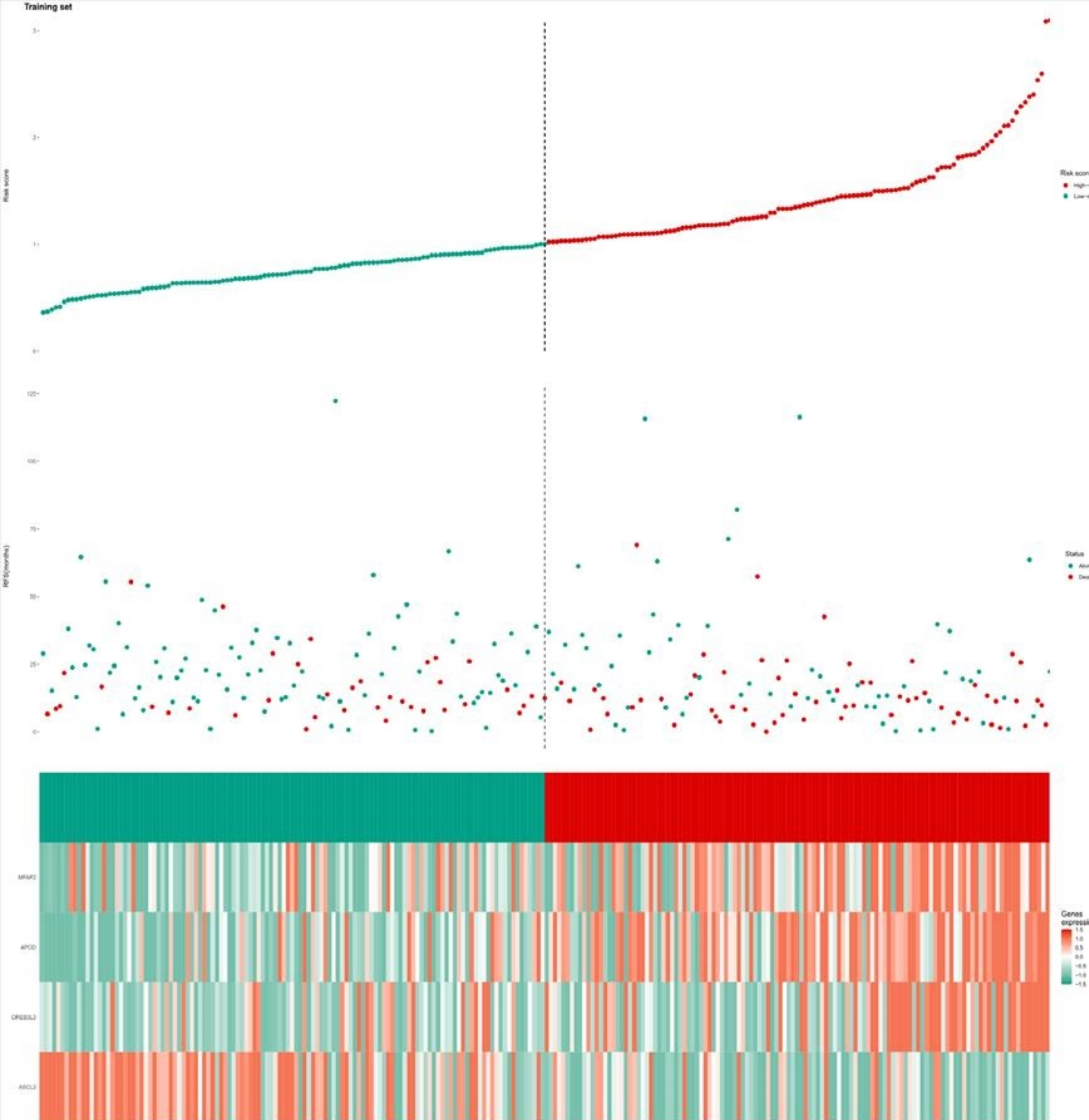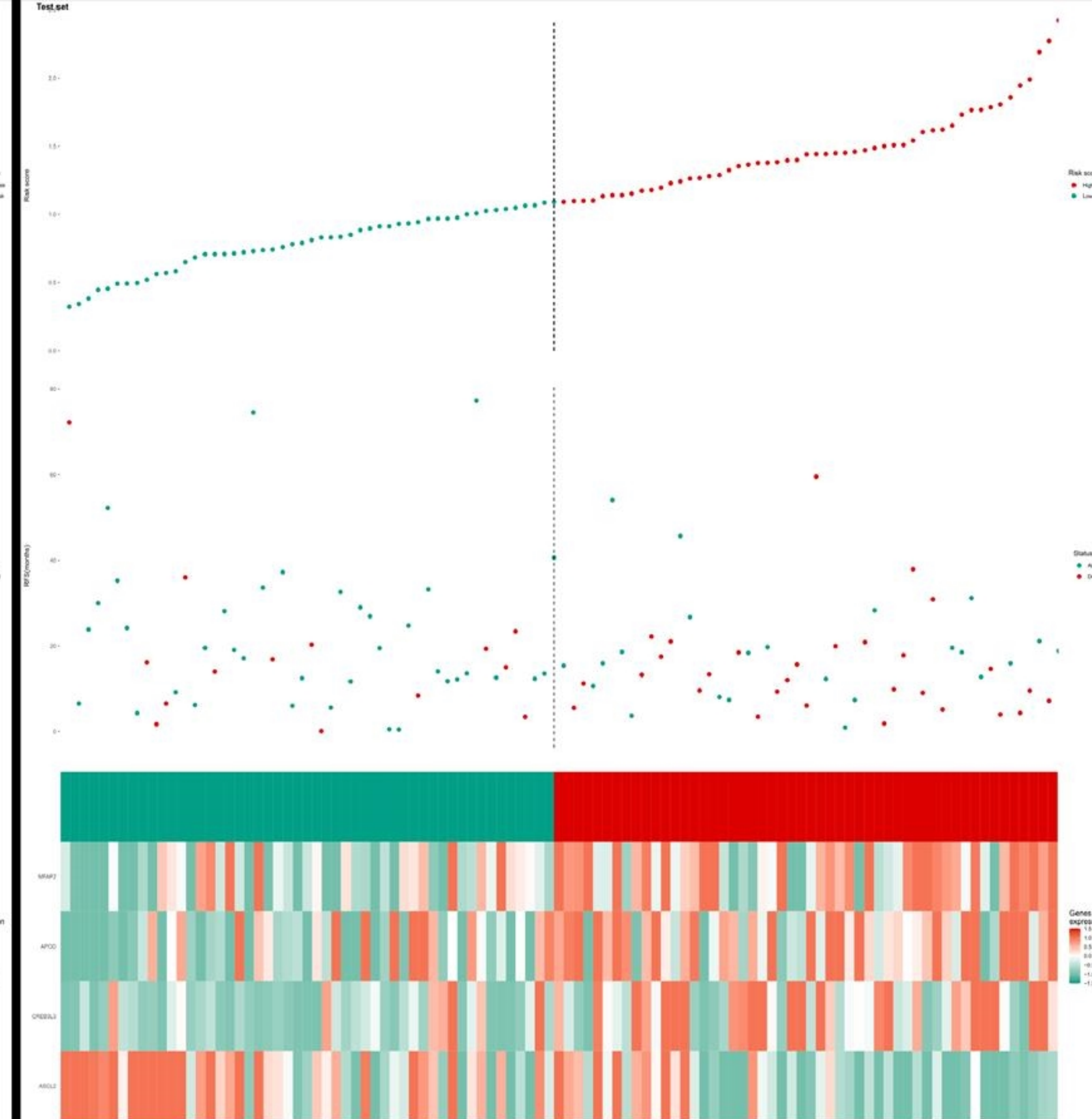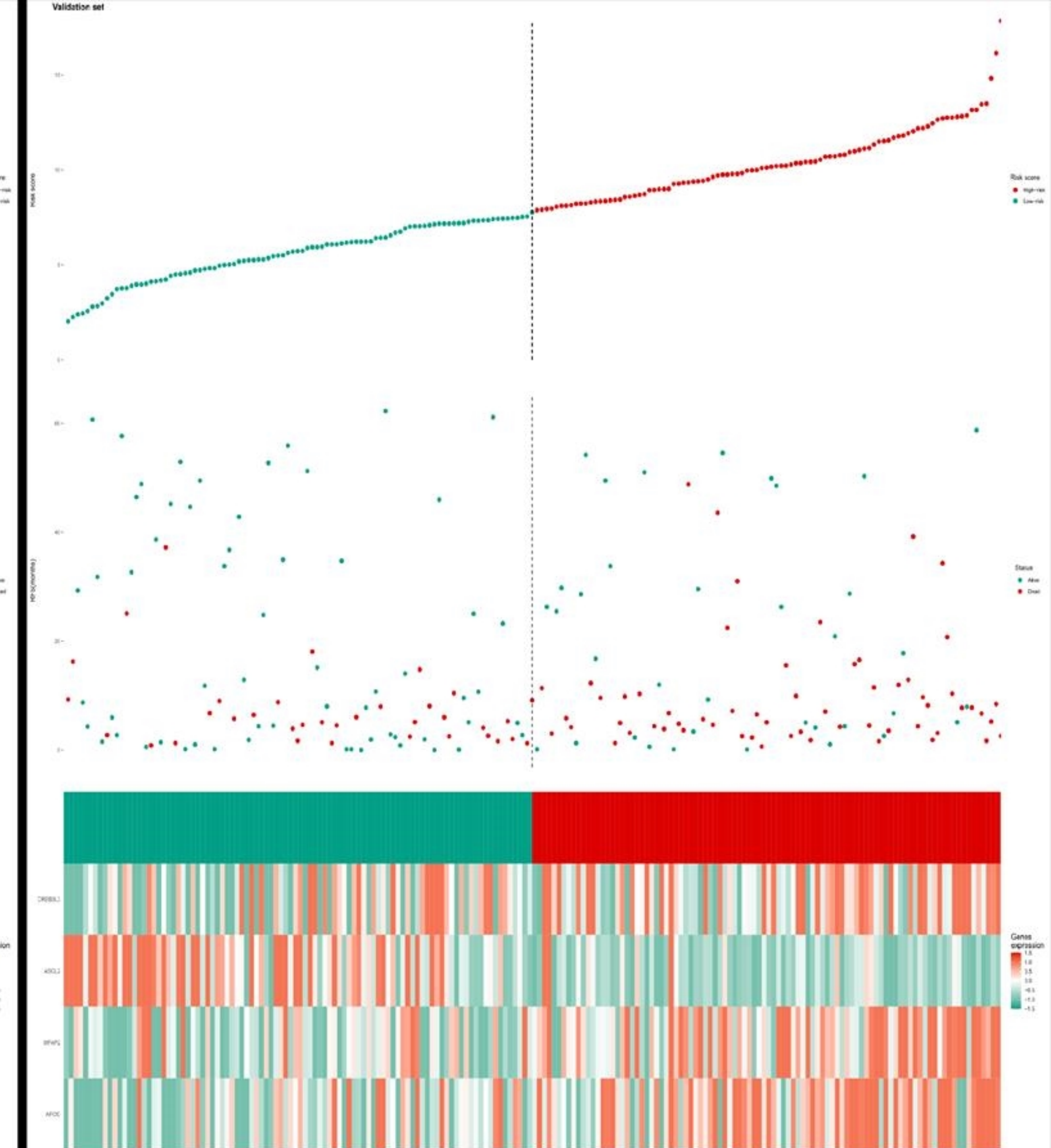

Supplement: Supplementary Materials — Supplementary Figure 1: determination of the k value using the NMF rank survey with multiple parameters. Supplementary Figure 2: OS and DSS analyses of different subtypes. (a) Overall survival (OS) curves for 5 different subtypes. (b) Disease-specific survival (DSS) curves for 5 different subtypes. P < 0.05 showed statistically significant. Supplementary Figure 3: validation of the m5C-related risk model. (a) The KM survival curve of the high- and low-risk group in the validation set. (b) The KM survival curve of high- and low-risk group in the testing set. (c) The ROC curve and AUC for four signatures in the validation set. (d) The ROC curve and AUC of four signature in the test set. Supplementary Figure 4: the risk score, survival time, survival status, and expression of the four signatures in the training set (a), validation set (b), and testing set (c). Supplementary Figure 5: KM survival stratification analyses between high- and low-risk GC samples with clinicopathological data. Supplementary Table 1: the primer sequences for qRT-PCR. Supplementary Table 2: the detailed information for TIDE analysis. [file 9931419.f1.zip › Supplementary Figure 4.pdf]

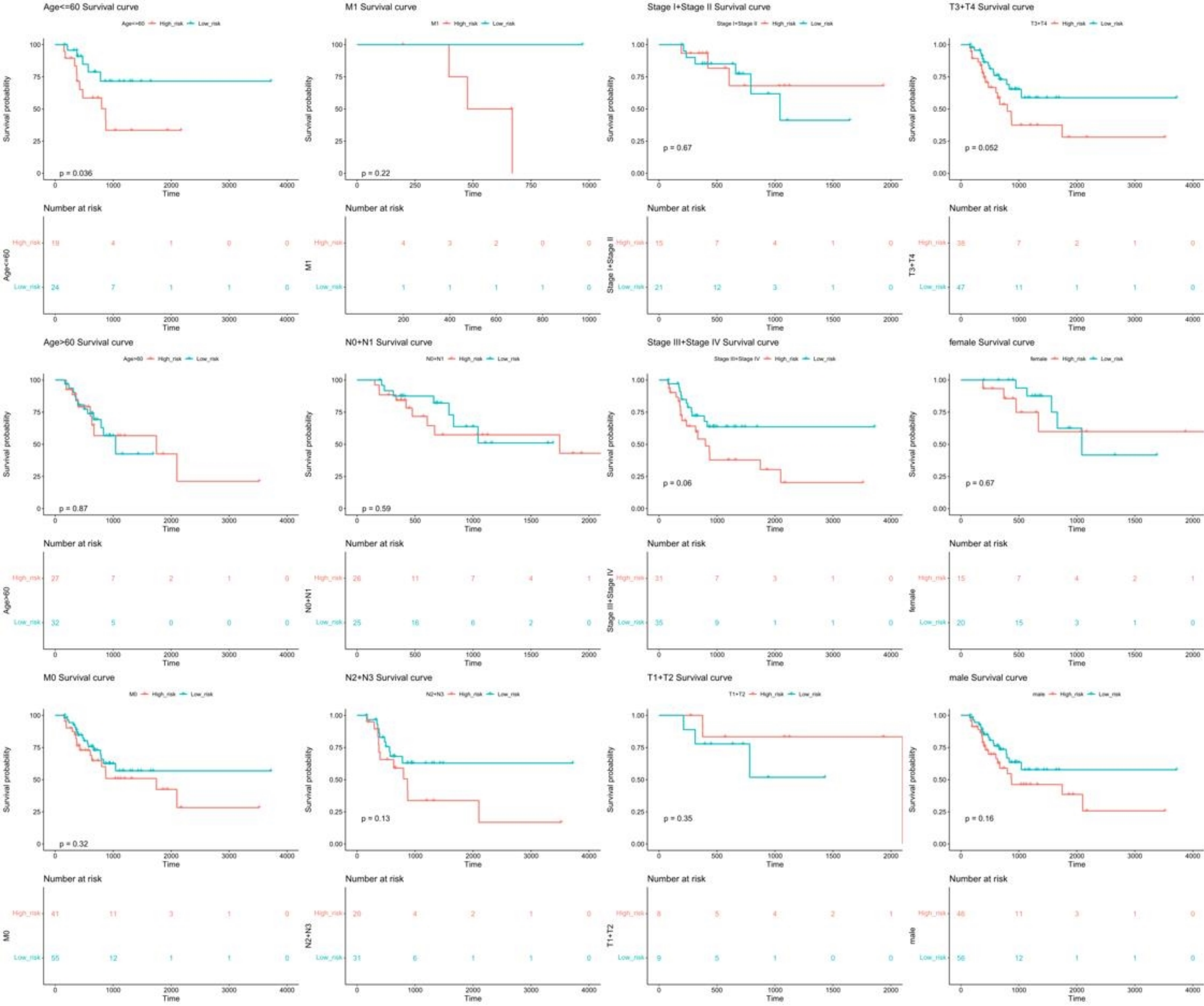

Supplement: Supplementary Materials — Supplementary Figure 1: determination of the k value using the NMF rank survey with multiple parameters. Supplementary Figure 2: OS and DSS analyses of different subtypes. (a) Overall survival (OS) curves for 5 different subtypes. (b) Disease-specific survival (DSS) curves for 5 different subtypes. P < 0.05 showed statistically significant. Supplementary Figure 3: validation of the m5C-related risk model. (a) The KM survival curve of the high- and low-risk group in the validation set. (b) The KM survival curve of high- and low-risk group in the testing set. (c) The ROC curve and AUC for four signatures in the validation set. (d) The ROC curve and AUC of four signature in the test set. Supplementary Figure 4: the risk score, survival time, survival status, and expression of the four signatures in the training set (a), validation set (b), and testing set (c). Supplementary Figure 5: KM survival stratification analyses between high- and low-risk GC samples with clinicopathological data. Supplementary Table 1: the primer sequences for qRT-PCR. Supplementary Table 2: the detailed information for TIDE analysis. [file 9931419.f1.zip › Supplementary Figure 5.pdf]
